# Aptamers: Design, Theory, and Applications to Diagnosis and Therapy for Diseases

**DOI:** 10.1002/mco2.70180

**Published:** 2025-05-19

**Authors:** Sepideh Hassibian, Mahsa Amin, Seyed Mohammad Taghdisi, Elham Sameiyan, Reza Ghaffari, Mona Alibolandi, Mohammad Ramezani, Khalil Abnous, Seyed Mohsen Dehnavi

**Affiliations:** ^1^ Department of Cell and Molecular Biology Faculty of Life Science and Biotechnology Shahid Beheshti University Tehran Iran; ^2^ Targeted Drug Delivery Research Center Pharmaceutical Technology Institute Mashhad University of Medical Sciences Mashhad Iran; ^3^ Department of Pharmaceutical Biotechnology School of Pharmacy Mashhad University of Medical Sciences Mashhad Iran; ^4^ Pharmaceutical Research Center Pharmaceutical Technology Institute Mashhad University of Medical Sciences Mashhad Iran

**Keywords:** antidote, aptamer, reversible therapeutics

## Abstract

Single‐stranded DNA or RNA entities referred to as aptamers exhibit a strong affinity and specificity for attaching to specific targets. Owing to their special properties, which include simplicity of synthesis, low immunogenicity, and adaptability in targeting a variety of substances, these synthetic oligonucleotides have garnered a lot of interest. The function of aptamers can be altered by combining them with complementary oligonucleotides “antidotes,” which are antisense to a particular aptamer sequence. Antidotes play an important role in several fields by specifically targeting the corresponding section of the aptamer. Nevertheless, even with their promising capabilities, the creation of antidotes to regulate or inhibit aptamer function continues to be a relatively unexamined field, constraining their secure and efficient application in medical environments. The review explores experimental methodologies for creating antidotes, the systematic design strategies for managing antidotes in aptamer‐based therapies, and their therapeutic efficacy in counteracting disease biomarkers. Additionally, it highlights their diagnostic applications in biosensing and imaging, offering a promising alternative to traditional antibodies. It also investigates the progress, latest innovations, and potential medical uses of aptamer–antidote combinations. Its academic value lies in bridging the gap between theoretical design and practical applications, providing researchers and clinicians with a comprehensive resource to advance aptamer‐based solutions in medicine and biotechnology.

## Graphical abstract

A summary showing potential applications of nucleic acid aptamers and antidotes. Aptamers and antidotes can be utilized in biomedical diverse areas. Nucleic acid aptamers have applications in several biomedical disciplines including theragnostic, molecular imaging, drug delivery, biosensors, and gene therapy. The therapeutic utilizes of aptamers can be classified into antagonists (inhibiting targets), agonists (activating targets), delivery systems, and gene therapy.

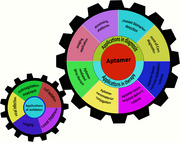

## 1 Introduction

Despite the current advances in clinical and preclinical strategies, cardiovascular and cancer diseases are still the leading cause of mortality worldwide [1]. RNA and DNA‐based medications are now recognized as a vastly growing field with promising applications in the diagnosis and treatment of an extensive range of health disorders, as well as for the management of risk factors [2]. Among these novel strategies, utilizing controllable therapeutic agents and the blockage of their effect can considerably reduce therapeutics’ harsh side effects [3].

Aptamers (Apts) consist of short single‐stranded sequences of DNA or RNA that are chosen from randomized and combinatorial libraries of oligonucleotides through the established in vitro selection technique known as SELEX [4]. Apt research has exploded over the last three decades, attributed to its distinctive features such as elevated specificity and binding affinity, reduced immunogenicity and toxicity, as well as the straightforwardness of synthesis that ensures minimal variation across batches [5]. Apts possess the ability to selectively attach to a variety of targets, which can include both small molecules and intricate structures, thus rendering them advantageous for numerous diagnostic and therapeutic uses. Apts function by adopting specific Three‐Dimensional (3D) configurations that facilitate binding to a target [6]. They are generated through a process called SELEX, where a randomized library of DNA or RNA is iteratively exposed to a target of interest until Apts with target specificity are identified [7]. Similar to antibodies, Apts achieve high levels of specificity for their target and strong binding affinities through the creation of complementary 3D structures, which can be integrated into the target [8].

Apts are employed as molecular probes in analytical scenarios rather than antibodies [9]. They are often utilized to identify diseases, microbes, viruses, environmental contaminants, or biomarkers [10]. Apts can be further developed for therapeutic purposes through chemical stabilization and modification procedures, extending their shelf life and serum half‐life [11]. The in vitro selection procedure has thus far produced a significant quantity of Apts or conjugates based on Apts [12]. Nonetheless, only a limited number of Apts have reached the market thus far, and various challenges hinder the successful clinical application of these Apts. The investigation into Apts remains in its early stage, and additional understanding of target interactions, tissue distribution, pharmacokinetics, and nucleic acid chemistry is essential [13].

**FIGURE 1:**
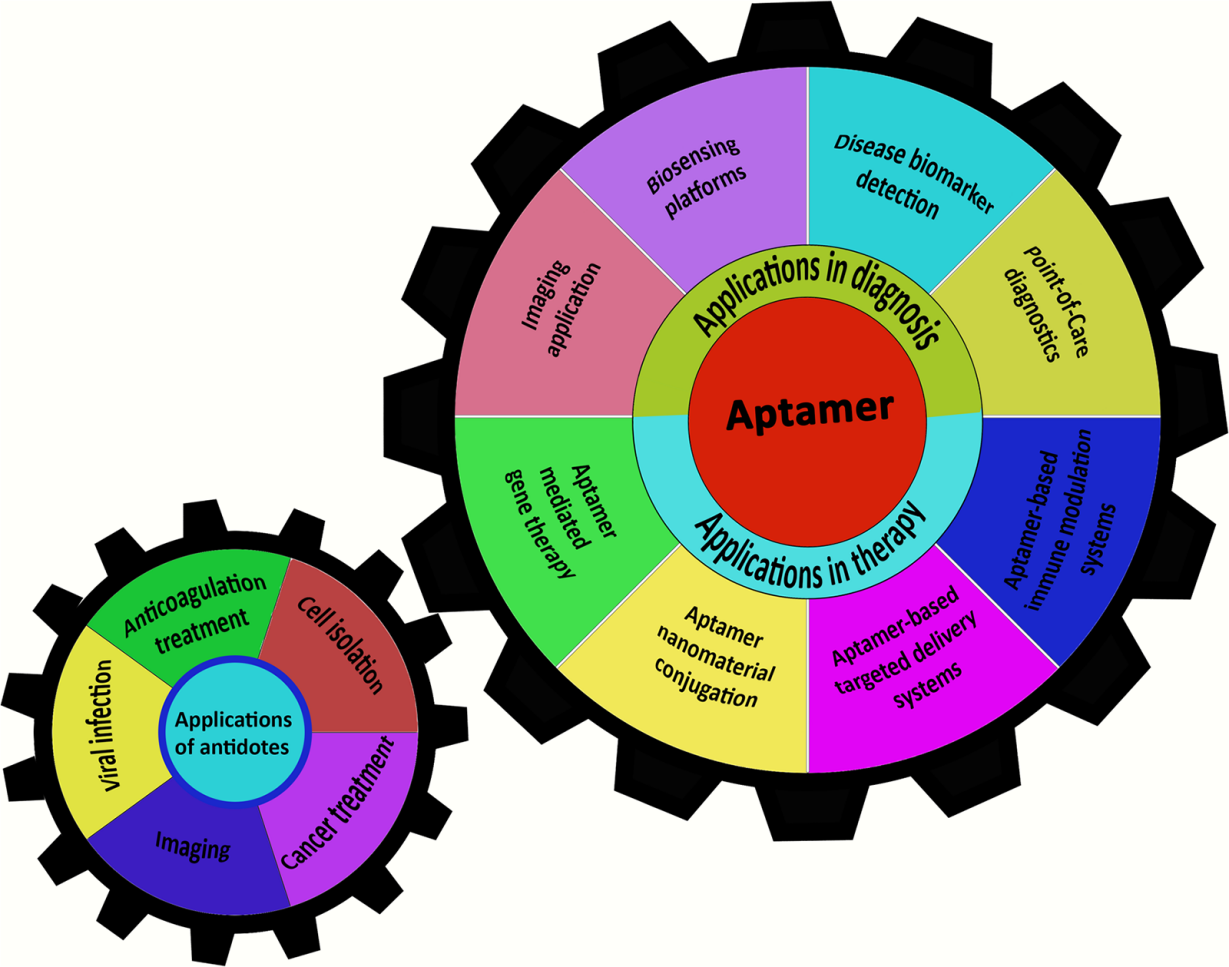
Various application fields of aptamers and antidotes. Aptamers and antidotes can be utilized in diverse biomedical areas. Nucleic acid‐based aptamers have applications in several biomedical disciplines including biosensors, molecular imaging, drug delivery and gene therapy.

Apts utilize secondary and tertiary structures to create matching surfaces. The formation of these structures is primarily influenced by interactions between Apt bases, meaning that introducing oligonucleotides that disrupt base pairing with certain parts of the Apt sequence can alter target binding [14]. This characteristic has been applied to generate numerous quickly reversible inhibitory Apts directed toward medically relevant proteins, potentially aiding in overcoming the obstacle of developing treatments [15]. The function of Apts can be altered by combining them with complementary oligonucleotides “antidotes” that are antisense to a particular Apt sequence [16]. Antidotes play an important role in several fields such as disease treatment, cell isolation, and animal imaging by specifically targeting the corresponding section of the Apt (Figure 1). Antidotes exert their effects by altering how the Apt behaves, transforming its conformational or improving its elimination [17]. This may also include disruption in how the Apt engages with its receptors, resulting in improved results in numerous instances. Hence, it is feasible to strategically control the functions of Apts to enhance the predictability of therapeutics [18]. This review emphasizes the Apt molecule along with the different strategies that can modulate the functionality of Apts within a medical framework. However, the antidote has several drawbacks. First, the development of customized oligonucleotide antidotes appropriate for each therapeutic Apt is costly. Second, it is difficult to develop an antidote for highly structured Apts. Ultimately, the subsequent Apt–antidote complex comprises a double‐stranded RNA or DNA, which has the potential to initiate an innate immune response and induce inflammation.

## 2 Structure and Properties of Apts

Apts are short oligonucleotide chains that possess a remarkable capability to recognize and bind to their specific targets with high affinity [19]. Apts are classified into two distinct categories according to their structural composition: peptide and nucleic acid. The term “aptamer” derives from the Latin word “aptus” (to fit) and the Greek word “meros” (particle), reflecting their role as ligands. The inherent flexibility of Apts allows them to encircle small molecular targets or to accommodate themselves within indentations and crevices on the surfaces of significantly larger target molecules [6]. Apts have the potential to bind to a wide range of targets, which includes peptides, proteins, small molecules, metal ions, chemical substances, as well as biological entities such as mammalian cells, viruses, bacteria, and yeast [20]. This characteristic arises from their innovative three‐dimensional folding, which facilitates outstanding binding selectivity [21]. The physical engagement between Apts and their respective targets leads to significant alterations in their structure, with the binding mechanism being facilitated by shape complementarity, hydrogen bonds, van der Waals interactions, electrostatic forces, and the stacking of planar groups [22].

In recent decades, Apts have become increasingly significant due to their higher effectiveness in delivery and sensing systems compared with monoclonal antibodies [23]. Their unique characteristics are the main factors that contribute to their competency. Thus, the next sections will outline the remarkable features of Apts as well as how they are generated.

In general, Apts are not identified as foreign substances by the human immune system, which allows them to be regarded as less immunogenic or nonimmunogenic, and nontoxic [24]. Apts exhibit enhanced thermal resilience, which provides stability to their formation. Their capacity to undergo chemical modifications through various methods is among their most advantageous characteristics, which helps to reduce renal filtration and enhance resilience against nuclease degradation [25]. We can further improve stability while maintaining distinctiveness and compatibility by utilizing targeted methods, which include chemically modifying the phosphate backbone, employing sulfur to remove oxygen from the phosphodiester bond and ribose component, capping the 3′ end with inverted thymidine, altering the 2′ position of the ribose sugar, and dimerizing the Apt molecules [26].

Apts possess a characteristic known as “reversible denaturation.” Due to this capability, they can be readily reinstated within a brief timeframe following thermal denaturation [27]. Additionally, this trait facilitates the ongoing use of Apts while minimizing decontamination costs [28, 29]. Further, Apts can be produced in just a few days, in contrast to the months required for monoclonal antibodies [30]. This leads to increased production speed and reduced costs. Apts are cheaper to produce, have a longer shelf life and their production scale is significantly larger than that of antibodies [31].

### 2.1 SELEX: Apt Synthesis Art

Apts can be produced in large volumes because of their minimal activity degradation while being stored, as well as their structural robustness [32]. In 2007, Stoltenberg et al. [33] utilized the term “Systematic Evolution of Ligands by EXponential enrichment” (SELEX) to describe the Apt in vitro synthesis. An oligonucleotide library typically comprises 50 to 90 single‐stranded randomized nucleotide sequences, each featuring primer binding sites at both termini. The process of Apt production involves several stages: (a) creating a random library containing 10^14^ to 10^16^ single‐stranded oligonucleotides and permitting these oligonucleotides to interact with their target ligand, (b) isolating the oligonucleotides that are bound from those that remain unbound, (c) selecting specific oligonucleotides and amplifying them through Polymerase Chain Reaction (PCR) (for DNA Apts), and (d) finally characterizing of the Apts through sequencing. This selection cycle is repeated until a sequence with the desired affinity is obtained [34]. Up to now, the main methods for identifying Apts have largely remained the same. Nonetheless, notable progress has been achieved in the characterization of Apts throughout the selection phase, the types of targets that can be examined, and the modifications that Apts can undergo, thereby broadening the practical uses of Apts in both in vitro and in vivo settings [35].

To create an RNA Apt library, a library of single‐stranded DNA that includes the T7 RNA polymerase promoter sequence at the 5′ end is prepared. To produce RNA Apts, the single‐stranded DNA library undergoes conversion into double‐stranded DNA, followed by in vitro transcription [36]. This entire procedure is reiterated until the target oligonucleotide (or Apt) exhibiting strong binding affinity is produced. Once the desired clones are isolated, they undergo further optimization to enhance their functionality [29]. They are reduced in size to achieve the minimal possible length for Apts while preserving the greatest binding affinity to the target. Apts typically have an ideal length of 15–45 nucleotides and possess a molecular weight of around 5–15 kDa. The binding affinities of Apts vary from pico to micromolar, and the SELEX methodologies have advanced to fulfill specific needs [37].

## 3 Applications of Apts

Apts exhibit a variety of notable characteristics, granting them a distinctive functionality and showcasing their extraordinary adaptability across a wide array of applications [15]. This section will focus on the various applications of Apts across several areas of interest (Scheme 2).

### 3.1 Applications in Diagnosis

It is widely recognized that timely identification of a disease plays a crucial role in determining its prognosis. Although factors such as age and additional risk elements can affect the choice of treatment, obtaining an early and accurate diagnosis is vital for effective therapy [38]. Timely identification is crucial for hindering the progression of diseases. As the amount of targets for identification diminishes, the diagnostic approach needs to enhance its sensitivity [39]. An effective diagnosis necessitates two fundamental conditions: the identification of irregular characteristics and accurate detection. Given that efficient methods for recognizing and detecting targets are crucial, recent initiatives have concentrated on the application of Apts. Apts serve as optimal targeting agents, thanks to their ease of functionalization through the attachment of a signaling agent, which facilitates precise detection. These additional factors often need to be considered to ascertain the binding and amplification of the signal. Depending on the chemical composition, size, charge, and the extensive range of pH and temperature resilience, modifications to Apts can be implemented without compromising their binding abilities. Thus, Apts and functionalization techniques are vital elements in formulating a sensitive diagnostic strategy, which requires considerable effort in both strategy formulation and probe design.

#### 3.1.1 Biosensing Platforms

Developing of a biosensing platform that is reliable, effective, simple, and affordable is essential for bioanalytical applications in various of fields, including clinical diagnosis, food safety, scientific research, and environmental monitoring. Given that the currently accessible biosensors do not meet the heightened demands for discovery, it is essential to extensively develop innovative biological instruments to establish a unified biosensing platform. This initiative should expedite the creation of diverse biosensor designs [40, 41, 42].

The emergence of infectious virus outbreaks presents considerable difficulties for global public health. The severe acute respiratory syndrome coronavirus 2 (SARS‐CoV‐2) is most notably responsible for the coronavirus disease 2019 (COVID‐19), resulting in a global pandemic and posing a significant risk to human health. There exists an urgent requirement for a method that allows for the detection of SARS‐CoV‐2 with high efficiency and swift sensing capabilities. In 2024, Ma et al. [43] reported the development of the inaugural instance of swift detection of SARS‐CoV‐2 antigen through the use of carbon‐nanotube‐array‐based thin‐film transistor (CNT‐array‐based TFT) biosensors integrating triple Apts and tetrahedral DNA nanostructures (TDNs). Based on their sensing mechanism, TFT biosensors employing CNT arrays are capable of identifying SARS‐CoV‐2 receptor‐binding domains (RBDs) with a response that surpasses that of CNT‐network‐based TFT and metal‐electrode‐based CNT–TFT biosensors by as much as 102%. The aptasensor could attain a low limit of detection (LOD) of 10 aM (6 copies/µL), allowing for the identification of wildtype SARS‐CoV‐2 RBD across an extensive detection range that covers eight orders of magnitude, achieved by integrating TDNs with triple Apts. This success can be attributed to the improved efficiency in protein capture. Hence, the amalgamation of TDNs, triple Apts, and CNT‐array‐based TFT biosensors provides a swift and high‐performance method for the detection of SARS‐CoV‐2. The adaptability of the aptasensor, facilitated through the modification of specific Apts, makes it feasible to rapidly detect the virus.

Electrochemical Apt‐based biosensors (E‐AB) possess significant potential for a variety of applications, such as drug monitoring. While many encouraging findings emerge from laboratory experiments, the challenge of converting these discoveries into marketable products persists due to issues related to insufficient durability in practical environments, the labor‐intensive preprocessing required for conventional electrodes, and the elevated costs associated with scaling up production. Liu et al. [44] developed an effective and economical E‐AB biosensor capable of detecting tobramycin, an aminoglycoside antibiotic, by utilizing printed circuit board (PCB) electrodes along with a compact and wireless electrochemical reader. They also employed the swift prototyping features of PCB technology to continuously alter the electrode configuration, resulting in optimal function. The suggested approach detects the intended drug by analyzing the alterations in electron transfer kinetics caused by binding‐induced conformational changes in Apts tagged with methylene blue. A complete assessment of the sensing technique was performed under controlled buffer conditions. The sensor exhibited a linear range extending from 750 µM to 10 mM, with a LOD of 125 µM. Furthermore, the sensor's functionality was effectively assessed in a matrix with interferences and across various temperatures and storage conditions, thereby validating its potential for commercial application.

#### 3.1.2 Disease Biomarker Detection

Timely identification of diseases is crucial for effective treatment, with serum biomarker assessments, such as enzyme‐linked immunosorbent assays (ELISA), representing the most common diagnostic method [45]. Nevertheless, the intricate makeup of serum and plasma poses challenges in identifying target biomarkers at low levels, thereby impeding early diagnosis. Certain techniques are also notably labor intensive and require considerable time [46]. Consequently, because of their exceptional sensitivity and specificity for targets present in minimal concentrations, Apts have surfaced as an innovative and encouraging means for the diagnosis of diseases. In contrast to antibodies, the structure of Apts changes upon binding to their respective targets [6]. This alteration in conformation presents the opportunity to develop distinctive, switchable probes based on Apts, which are undetectable by antibodies [47].

Dementia is a neurological disorder that leads to a lasting and advancing decline in both cognitive abilities and motor skills. In spite of worldwide initiatives, there remains no straightforward and dependable means for diagnosing or treating this condition. Present diagnostic approaches involve indirect assessments of typically difficult‐to‐obtain biological fluids and low‐resolution imaging of the brain. In 2023, Bodily et al. [48] developed a compact, wireless biosensor platform utilizing a readout‐based graphene field‐effect transistor (GFET), which is capable of identifying small molecules and pathogens with single‐molecule precision and sensitivity. They demonstrated the identification of three significant amyloids, specifically, amyloid beta (Aβ), Tau (τ), and α‐synuclein (αS) through the use of DNA Apt nanoprobes. To achieve this, they assessed the performance of the GFET platform by utilizing atomic force microscopy, Raman spectroscopy, and electrical measurements. They modified the graphene surface by attaching well‐known high‐affinity Apts that are specific to a range of proteins associated with neurodegenerative diseases, namely Aβ1–42, Tau441, and αS. These proteins were characterized, isolated, and purified from the brain tissues of patients who had undergone autopsy due to Alzheimer's disease (AD) and Parkinson's disease (PD). In the subsequent phase, they assessed the selectivity and LOD of the aptasensor concerning these amyloid proteins by utilizing synthetic isoforms within a regulated buffer setting. Furthermore, they tested the system against amyloid proteins derived from the brain to develop a dependable sensor for identifying amyloid protein biomarkers in samples from AD patients. By conducting appropriate control experiments, they illustrated notable specificity and minimal cross‐reactivity. In summary, their results indicate that the Apt‐GFET sensor is capable of precisely detecting protein biomarkers associated with AD and PD.

Fluctuations in the levels of luteinizing hormone (LH) have considerable impacts on the entire reproductive cycle and regulation of the meiotic cell cycle of oocytes [49]. Hence, it is crucial to sensitively measure the LH biomarker to effectively diagnose reproductive disorders. In 2024, Ming et al. [50] developed an exceptionally sensitive bio‐electrochemical Apt LH detection technique, which uses utilizes an innovative low‐background catalytic redox recycling mechanism alongside a hybridization chain reaction (HCR). The creation of HCR involving [Ru(NH_3_)_6_]Cl_3_ (RuHex)‐modified dsDNA polymers on the sensor electrode starts when LH analyte molecules release single strand DNAs (ssDNAs) from the Apt strands within the duplex DNAs. Thus, the electrochemical redox recycling of RuHex, facilitated by K_3_[Fe(CN)_6_], results in markedly enhanced currents for the highly sensitive detection of LH. Low background redox recycling and HCR signal enhancement operate together to yield a significantly enhanced signal‐to‐noise ratio and sensitivity for the detection of LH at concentrations as minimal as 6.03 pM. Hence, they established a reliable detection framework for detecting various biomarkers at low concentrations, facilitating early disease diagnosis. Furthermore, the detection of LH in diluted human serum has also been explored and confirmed.

It has been shown that exosomes originating from cancer, serving as indicators in liquid biopsies, play a crucial role in the early detection, diagnosis, and prognosis of cancer. However, the current detection approaches face challenges such as extended time requirements and inadequate sensitivity. To achieve the identification of tumor‐derived exosomes with remarkable sensitivity and without any preprocessing steps, Pan et al. [51] developed a sandwich‐type electrochemical sensing apparatus utilizing Prussian blue/graphene oxide (GO/PB) along with spiky Au@Fe_3_O_4_ nanoparticles (NPs) for the determination of MCF‐7 exosomes. This approach bypassed the extraction and purification steps that were characteristic of earlier recognition methods by employing nanospike structures on magnetic beads to produce spiky Au@Fe_3_O_4_. This material was subsequently employed to concentrate exosomes from serum samples. The enrichment of spiky Au@Fe_3_O_4_, along with signal amplification, can also considerably enhance the detection sensitivity of the sensing method. GO/PB was employed to immobilize antibodies aimed at recognizing and attaching to the captured exosomes, thereby generating electrochemical signals. The MUC1 protein was adopted as the detection target because of its overexpression in MCF‐7 exosomes. Thus, a highly sensitive electrochemical biosensing method was successfully developed to determine exosomes with a low LOD. The constructed electrochemical sensor exhibited exceptional performance in identifying MCF‐7 exosomes, paving the way for innovative strategies in future cancer diagnostics and assessing the expression of surface proteins on exosomes.

Glaucoma ranks as one of the foremost contributors to irreversible blindness globally. Timely identification coupled with swift treatment plays a crucial role in influencing the outcome. Precise tracking of the biomarker Growth/Differentiation Factor 15 (GDF15) aids in the correct diagnosis and assessment of glaucoma. In 2023, Gao et al. [52] found that the observation of GDF15 levels can aid in the assessment of glaucoma risk and facilitate early detection. They engineered an Apt (APT2TM) characterized by high selectivity, affinity, and stability for attaching to GDF15 in both humans and rats. Simulation studies indicated that the interplay between van der Waals forces and polar solvation energy significantly influenced the binding properties of APT2TM, with salt bridges and hydrogen bonds playing crucial roles. They later integrated an enzyme‐linked Apt sandwich assay (ELASA) with a biolayer interferometry (BLI) system to create a high‐throughput, automated, real‐time system known as BLI–ELASA. This approach featured a wide dynamic detection range (10–810 pg/mL) and exceptional sensitivity for GDF15. Additionally, they observed that it functioned effectively when utilized to measure GDF15 in actual specimens from glaucomatous rats and clinical human subjects. They also believe that this method would offer a straightforward, robust, and cost‐effective approach for screening, early detection, and evaluation of animal models of glaucoma in the years ahead.

#### 3.1.3 Imaging Application

Apts have arisen as powerful imaging agents because of their distinctive characteristics. Among these are their small size and poly‐ionic composition, which may facilitate the swift elimination from the bloodstream and absorption by tissues [53, 54]. This could help to shorten the time spent in the liver and kidneys. Apts are regarded as nonimmunogenic and demonstrate superior functionality owing to their straightforward chemical modification capabilities. In addition, the ability to make flexible alterations at both the 3′ and 5′ ends, combined with their high specificity and affinity, renders them excellent candidates for use as imaging agents across different imaging modalities [55].

Currently, cancer stands as one of the leading causes of death and is a prevalent illness worldwide [56, 57]. Timely and accurate diagnosis of cancer is crucial for enhancing the survival rates of patients [58]. Therefore, there is an urgent need for effective molecular ligands that can selectively detect cancer. In 2021, Li et al. [59] utilized a tissue‐based SELEX methodology to identify a new DNA Apt known as SW1. Cancerous liver tissue samples served as the positive control, while adjacent normal liver tissue samples were applied as the negative control. Employing immobilized liver cancer SMMC‐7721 cells as a research specimen, the Apt SW1 exhibited a strong affinity, with its binding target initially recognized as a non‐nucleic acid element within the nucleus. In addition, the data obtained from tissue imaging indicated that SW1 was particularly effective on identifying malignant liver tissues, achieving a high detection rate of 72.7%, while demonstrating a significantly lower detection rate for adjacent normal tissues. The Apt SW1 has proved its capability to detect different types of cancer cells and tissues, including those associated with liver cancer. Additionally, SW1‐A, an enhanced variant of SW1, maintained its strong binding affinity for liver cancer cells and tissues. In summary, our results indicate that SW1 holds considerable promise as a molecular tool for cancer detection in clinical settings.

The current state of activatable DNA biotechnology does not yet possess the capability to alter functional DNA (fDNA) through the use of proteases [60]. To address this limitation, Xiang et al. [61] introduced a conditional DNA Apt technology that is activated by proteases, allowing for tumor‐specific biosensing and imaging capabilities. The fDNA‐derived probe was developed by stabilizing the structural‐switching ability of an ATP Apt through the application of a peptide nucleic acid (PNA)–peptide–PNA triblock copolymer. They additionally illustrated that the protease was capable of selectively triggering ATP sensing functions in cancer cells, both in laboratory settings and within living organisms, resulting in enhanced specificity for tumors. The use of PNA‐directed fDNA engineering with peptides facilitates the functional modulation of any fDNA configuration, effectively connecting these two domains. This research demonstrates how connecting fDNA with peptides may lead to accurate biological applications.

#### 3.1.4 Point‐of‐Care Diagnostics

Point of care (POC) diagnosis refers to the process of conducting a systematic examination in close proximity to the patient, aimed at aiding in therapeutic and management choices [62, 63]. This form of diagnostics enables efficient treatment without incurring costs associated with laboratory testing [64]. POC tests for infections have significantly lowered mortality rates in developing nations by enabling precise diagnosis and offering treatment options [65, 66]. Thanks to their user‐friendliness, availability, rapid detection capabilities, high precision, and sensitivity, POC tests are advantageous during the ongoing epidemic and alleviate the testing demands placed on healthcare systems [67, 68].

The prompt identification of the human immunodeficiency virus‐1 (HIV) is crucial and can be achieved by employing its cell surface glycoprotein‐120 without necessitating any prior processing of the virus [69]. In 2023, Rizvi et al. [70] presented a POC method for detecting HIV directly in plasma samples by modifying both volume and color, while also enabling quantitative detection with minimal requirements. In order to reach this objective, they initially created a photonic crystals array (PCs‐array) through the self‐assembly of polystyrene NPs on the surface of water, which was subsequently transferred onto a glass slide. Subsequently, a polyacrylamide hydrogel was synthesized on the surface of the PCs array under ultraviolet light. In the final phase, a stable single‐stranded DNA Apt (modified with acrydite) was crosslinked within the polyacrylamide hydrogel that was integrated with PCs, utilizing ethylenediaminetetraacetic acid. The resulting APC‐hydrogel technique benefits from the properties of PCs, which are capable of diffracting visible light owing to the existence of photonic band gaps.

In 2016, Dirkzwager et al. [71] developed prototypes of a POC device aimed at diagnosing malaria through an Apt‐based colorimetric assessment for the recognition of Plasmodium falciparum lactate dehydrogenase using an Apt‐tethered enzyme capture (APTEC) assay. The use of 3D printing was instrumental in the assembly and optimization of these devices, enabling swift testing. A paper‐based syringe and a magnetic bead‐based well test were designed. Both tests demonstrated high sensitivity for recombinant Pf LDH as a previously mentioned 96‐well plate APTEC assay, yet they require a reduced sample volume (20 µL) and do not necessitate any additional tools or specialized knowledge for implementation. In this approach, the objectives of the research were achieved. While the syringe assay exhibited a broad dynamic range and enhanced analytical sensitivity, it necessitated further sample preparation techniques before assessing whole blood, thereby complicating the entire process. In contrast, the well test exhibited a restricted dynamic range; however, it successfully evaluated whole blood samples without any issues. Thus, at this preliminary phase of its development, the well test outperformed the syringe test as a comprehensive qualitative and user‐friendly diagnostic tool for POC applications, especially because the dry reagents could be stored within the well and sealed with foil.

The prompt identification of cardiovascular diseases (CVDs) necessitates dependable, rapid, and accurate assessments of plasma low‐density lipoprotein (LDL) at the time of diagnosis. While immuno‐sensors have often been employed for this objective, the complexities involved in sample preparation and the elevated costs represent significant drawbacks of this approach. In 2020 Wang et al. [72] created a sandwich‐style aptasensor featuring magnetic flexibility and utilizing disposable screen‐printed electrodes (SPE). At the outset, LDL present in plasma was swiftly captured and isolated using the Fe_3_O_4_@SiO_2_ complex. The LDL on Fe_3_O_4_@SiO_2_ was linked to an Apt (Apt) and a coimmobilized ferrocene (Fc) within the UiO‐66 metal–organic framework (MOF‐Fc@Apt) serving as a signaling marker. For the purpose of signal generation, the sandwich complex can be magnetically attached to SPE. This method has shown several distinct advantages. To effectively capture and isolate LDL from intricate plasma samples with sufficient selectivity, Fe_3_O_4_@SiO_2_ was first employed instead of expensive immunosorbent materials. In order to enhance both the sensitivity and specificity of detection, a considerable amount of Fc and Apt could be enclosed within MOF‐based signal tags. Furthermore, the magnet swiftly adsorbed the LDL sandwich complex linked with the composite probes onto the SPE for the purpose of signal generation, and this complex was promptly removed through washing. Hence, the measuring platform could therefore be used again. Also, the sample amount was only 0.1 mL, and the detection process took only 20 min in total. Due to these characteristics, the assay was an excellent candidate for LDL POC testing with the goal to detect CVD early.

### 3.2 Applications in Therapy

In the early years of the 20th century, Dr Paul Ehrlich introduced the concept of the “magic bullet” in relation to cancer treatment, asserting that optimal therapeutic agents would solely destroy cancer cells that were precisely identified as targets [73]. Modern medicine has continuously attempted to prevent undesirable side effects by selectively delivering medications to certain cells or tissues. Additionally, the widespread systemic administration of pharmaceuticals, which may negatively influence the bioavailability or concentration within affected regions, represents a significant challenge in conventional medicine. Moreover, systemic delivery entails that drugs traverse the bloodstream and affect the function of healthy tissues, potentially resulting in unavoidable adverse effects [74]. In this regard, the development of Apt technology has sparked a number of innovative studies that have effectively modified the concept of “targeted therapy” into the design of innovative “on‐demand” drug delivery systems. Apts have also been developed as the vehicles for targeted delivery tools conjugated to drugs, oligonucleotides, and NPs [75].

#### 3.2.1 Apt‐Based Targeted Delivery Systems

Targeted delivery, recognized as a crucial aspect of biomedical sciences, has frequently been utilized in the realms of pathological angiogenesis and oncology [76]. This approach can be executed through nanomaterial–Apt conjugations or Apt–drug conjugates (ADCs). The first takes advantage of the extensive surface area of NPs for incorporating diverse chemotherapeutic agents, in addition to their possible therapeutic effects, including the generation of reactive oxygen species (ROS). The latter is primarily utilized for administering intercalating agents such as proflavine, doxorubicin (DOX), and daunomycin, which reduces the toxicity to healthy cells by targeting specific cancerous cells [77]. Furthermore, the integration of an Apt with therapeutic agents has the potential to enhance in vivo selectivity and sensitivity, diminish drug toxicity, and advance oncological imaging. Such systems are capable of detecting or forecasting cancer, in addition to obstructing cancer signaling pathways [78]. This section addresses studies have employed Apts in targeted‐delivery systems, and the resulting outcomes are illustrated in Table 1.

**TABLE 1:** A summary of aptamer‐based targeted delivery systems.

Method of delivery	Aptamer	Therapeutic agent	Marker	Cell or animal	References
Utilizing silver NPs functionalized with PEG and AS1411 aptamer for irradiation therapy	AS 1411 aptamer	Silver NPs	Nucleolin	C6 glioma‐bearing mice	[79]
Sgc8 aptamer‐modified silica NPs system	Sgc8 aptamer	Doxorubicin (DOX)	PTK‐7	Human acute T lymphocyte leukemia	[80]
SWNT/piperazine–polyethyleneimine conjugate EpCAM aptamer	EpCAM aptamer	siRNA for suppressing BCL9l	EpCAM	MCF‐7	[81]
Paclitaxel (PTX) covalently binds to the nucleolin AS1411 aptamer (NucA) through dipeptide bond	AS1411 aptamer	Paclitaxel (PTX)	Nucleolin	SKOV3 (ATCC HTB‐77), OVCAR3 (ATCC HTB‐161)	[82]
Silica‐coated Gd–Zn–Cu–In–S/ZnS QDs/PEG/EpCAM DNA	EpCAM aptamer	DOX	EpCAM	4T1, MCF‐7	[83]
Liposome‐loaded aptamer–DOX	Aptamer AS1411	DOX	Nucleolin	MCF‐7/Adr cells	[84]

#### 3.2.2 Apt–Nanomaterial Conjugation

Nanomaterials, regarded as a crucial element in targeted delivery systems, can be integrated with Apts to fulfill imaging goals and therapeutic needs [85]. These materials are viewed as a favorable choice for functionalization with Apts because of their biodegradability, substantial loading capacity, straightforward bioconjugation process, drug release capabilities, and excellent permeability. Targeted drug delivery to cancer cells and tissues can significantly minimize cytotoxicity while increasing therapeutic effectiveness [86, 87]. Moreover, because NP carriers react swiftly to environmental stimuli or signals within cells, it is possible to achieve a controlled release of pharmaceuticals. This specific form of targeted delivery capitalizes on various advantages, such as the extensive surface area of nanomaterials for Apt attachment, preservation of Apts against nuclease degradation, and remarkable selectivity and sensitivity [88]. The subsequent discussion will concentrate on NPs that are frequently utilized in drug delivery systems, including gold (Au) and silica NPs.

AuNPs exhibit distinct electrochemical and optical characteristics, a surface plasmon resonance effect, excellent biosafety, and straightforward bioconjugation through Au–S chemistry, making them practical in targeted delivery systems [89]. AuNPs can transport therapeutic and imaging substances to tumor sites. In 2024, Alikhani et al. [90] presented a nanocomplex designed for chemo‐photothermal treatment of melanoma. This nanocomplex consisted of Au nanocages (AuNC) that were enveloped in polydopamine (PDA), a polymer responsive to light, and were infused with DOX. The nanocomplex (AuNC–DOX–PDA) was linked to a Sgc8‐c DNA Apt to facilitate targeted delivery. The Sgc8‐c DNA Apt shows a considerable degree of specificity for Protein Tirosine Kinase 7 (PTK7) receptors, commonly present in various cancer cell types, especially melanoma. Multiple studies have shown that Sgc8‐c Apts possess high specificity and selectivity for the swift detection of melanoma. The formulation's near‐infrared (NIR)‐responsive release profile has been confirmed in vitro, indicating efficient photothermal conversion. In chemo‐Photothermal Therapy (PTT), the NIR‐responsive Apt–AuNC–DOX–PDA exhibited significantly greater cellular toxicity compared with alone chemotherapy, aligning with in vitro findings. Additionally, the nanoplatform has shown effectiveness in computed tomography imaging. These results underscore the remarkable customized theranostic capabilities of Apt–AuNC–DOX–PDA for integrated chemo‐PTT in the management of melanoma, along with its application in diagnostic imaging.

Epirubicin (Epi) is a chemotherapeutic drug frequently utilized in cancer treatment. Despite its efficacy, the usage of this medicine is diminishing, owing mostly to drug resistance in tumor cells and myocardiopathy. These adverse effects could potentially be alleviated through the use of targeted nanocomplexes. Yazdian et al. [91] conducted a study on the specific delivery of Epi to colon cancer cells utilizing Mucin‐1 (MUC1) Apt in conjunction with ferritin NPs (Ft NPs), leading to the creation of Apt–Epi Ft NPs. Subsequently, the physicochemical characteristics of the NPs were assessed, including parameters such as size, zeta potential, structural integrity, drug loading capacity, release profile, drug uptake by cancer cells, cytotoxic effects, and in vivo data were collected. The findings indicated that the NPs were produced with an average size of 37.9 nm and an encapsulation efficiency of 67%. The release profile of the NPs revealed approximately 90% within 4 h in an acidic environment. Furthermore, the targeted administration of Epi enhanced its anticancer effects both in vitro and in vivo.

In 2024 Abrishami et al. [92] developed a core–shell theranostic system based on superparamagnetic iron oxide NPs and mesoporous silica (SPION@MSNs). Upon the incorporation of Epi into the accessible pores of MSN, the plasmid that encodes antimiR‐21 (pDNA) enveloped the external surface aided by a ZIF‐8 (zeolitic imidazolate framework‐8) coating. The surface was subsequently altered using AS1411 Apt and polyethylene glycol (PEG) to improve the targeting, biocompatibility, and protective characteristics of the nanocarrier. In addition, the loading capacity, release profile, and physicochemical properties of pDNA and EPI were extensively assessed. In vitro investigations have been conducted to examine the absorption of NPs by colorectal cancer and healthy cell lines, along with the anticancer impacts linked to targeted combination therapy.

#### 3.2.3 Apt‐Based Immune Modulation Systems

Over the past 10 years, there has been a pressing demand for the creating innovative therapeutic strategies that can complement conventional chemotherapy in order to enhance its anticancer efficacy [93]. Furthermore, it is thought that the domain of immuno‐oncology is progressing toward a comprehensive strategy aimed at tackling tumors from various perspectives [94]. In response to the favorable outcomes observed in recent clinical trials utilizing immune checkpoint inhibitors, cancer immunotherapy has attracted considerable interest. The immune system is sophisticated, yet it often struggles to function effectively.

The absence of antigenicity and costimulatory signals, coupled with the existence of immune‐suppressive agents within the tumor microenvironment, facilitates immunological evasion. Hence, the primary obstacles in cancer immunotherapy consist of: stimulating the immune system to target the tumor by initiating costimulatory signals within the tumor, diminishing inhibitory signals to enhance immune system activity in the tumor microenvironment, and augmenting tumor antigenicity through the expression of strong tumor neoantigens [95]. Following this strategy, cancer could be attack from several aspects. Additionally, as noted recently, an insufficient presence of tumor antigens poses a considerable obstacle to the effectiveness of immune checkpoint modulators [96]. Pastor et al. [97] demonstrated an effective method to enhance the generation of new tumor antigens through the modulation of nonsense‐mediated mRNA decay suppression within the tumor environment. This approach facilitates the expression of mutated tumor antigens containing premature stop codons, which are typically suppressed by this mechanism. Numerous constructs of Apts that can modify the immune response to cancer have been documented thus far. Their efficacy is comparable to, or may even surpass, that of the corresponding monoclonal antibodies, and they exhibit fewer off‐target side effects due to their enhanced targeted delivery capabilities. Since Apts can be specifically engineered to either activate or inhibit an immune‐modulatory receptor, their adaptability positions them as a highly promising option for immune‐modulatory ligands. Additionally, they can be tailored to transport almost any type of payload and facilitate immunomodulation at the tumor site.

**FIGURE 2:**
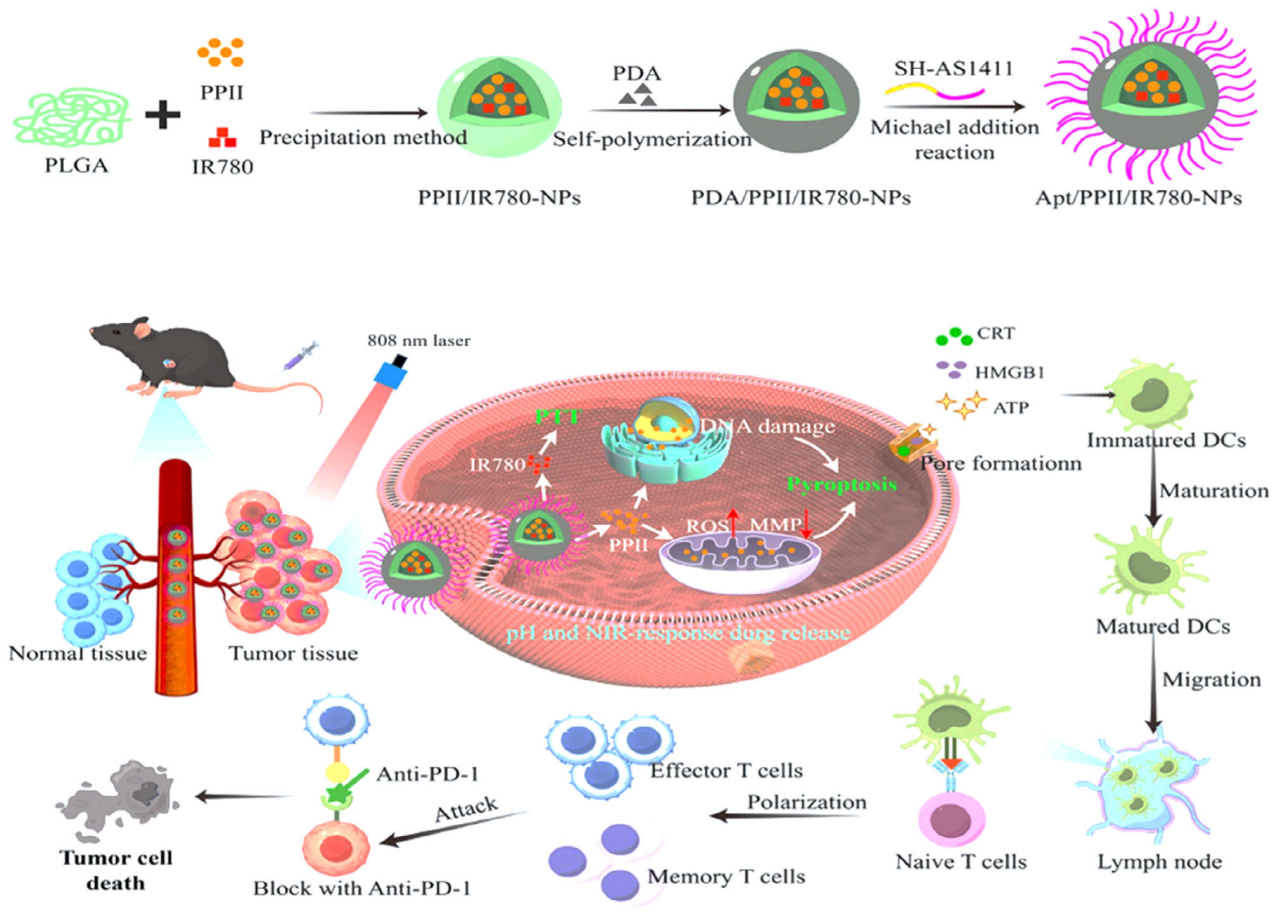
Pyroptosis combined with photothermal therapy. (A) Illustration of Apt/PPII/IR780‐NPs generation and (B) mechanism of enhancing liver cancer immunotherapy by inducing pyroptosis combined with photothermal therapy [98]. Copyright © 2024, Springer Nature.

In 2024, Huang et al. [98] introduced a method for the codelivery of IR780 polylactic‐co‐glycolic acid (PLGA) NPs alongside polyphyllin II, which promotes pyroptosis in combination with photothermal therapy to enhance antitumor immunity. To fulfill the multifaceted synergistic effect against liver cancer through immunotherapy, chemotherapy, photothermal treatment, and the pyroptosis inducer, the photothermal agent IR780 was transported to the tumor site using PLGA, as illustrated in Figure 2. The surface of the NPs was modified with Apt AS1411 to further refine their targeting capabilities toward tumors. Apt AS1411 is one of the initial Apts to advance to the clinical evaluation phase, demonstrating the ability to specifically attach to the overexpressed nucleolin receptors found on various tumor cell membranes. The active targeting and pH/NIR dual‐response release functionalities of NPs enhanced their antitumor efficacy while simultaneously minimizing the systemic toxicity associated with Polyphyllin II (PPII). These NPs, positioned in proximity to tumor cells, facilitated the generation of ROS and caused DNA damage, which culminated in pyroptosis. Subsequent to photothermal therapy, damage‐associated molecular patterns such as High Mobility Group Box 1 (HMGB1), Cardiac Resynchronization Therapy (CRT), and ATP are released, aiding in the maturation of dendritic cells. This procedure activates T cells, thereby boosting tumor‐specific adaptive immunity and establishing a robust immunological memory.

**FIGURE 3:**
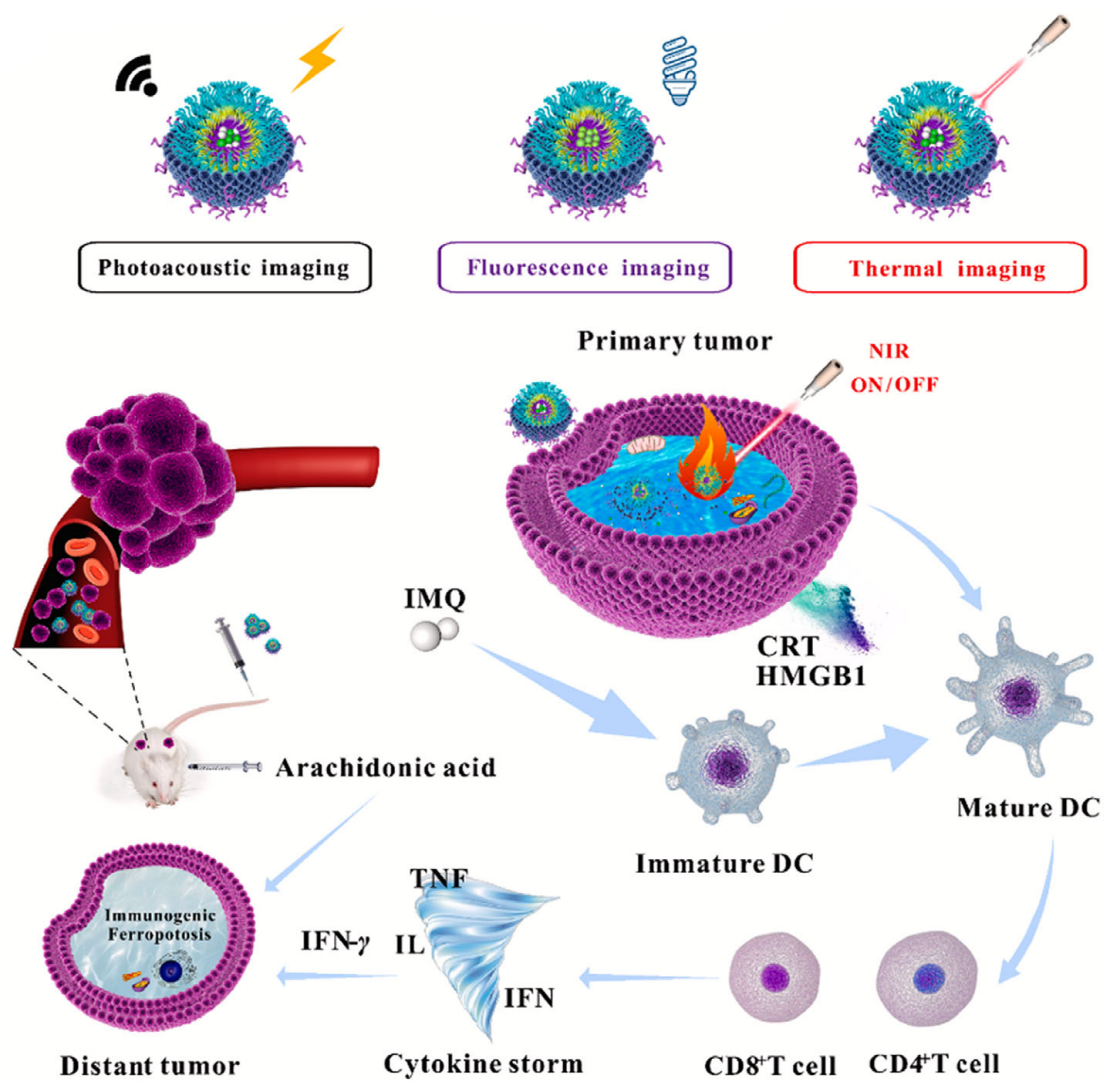
The role of EpCAM–CS‐co‐PNVCL@IR780/IM NPs in inducing antitumor immunity through ferroptosis. Under NIR irradiation, EpCAM–CS‐co‐PNVCL@IR780/IMQ NPs induce ICD in tumor cells, and activate CTLs that release cytokines [99]. Copyright 2025, Elsevier.

In another study, Xu et al. [99] created a nanoswitch activated by NIR light reacting to changes in pH and temperature. As illustrated in Figure 3, this nanocomplex was formed by initially merging chitosan (CS) and poly(N‐vinylcaprolactam) (PNVCL) through reversible addition‐fragmentation chain transfer polymerization. Following this, the epithelial cell adhesion molecule (EpCAM) Apt was linked to the CS‐co‐PNVCL polymer via an amide bond. The EpCAM Apt is commonly found to be overexpressed in breast tumor cells. Recent studies indicate that inhibiting EpCAM may be beneficial in the in vivo treatment of breast cancer. Amphiphilic block copolymers, such as those synthesized in this research, are recognized for their ability to spontaneously organize into nanomaterials when placed in buffered solutions. The creation of NPs facilitates the incorporation of therapeutic agents. The researchers opted to incorporate the IR780, which has been employed for tumor photothermal therapy and photoacoustic imaging, into the NPs. IR780 absorbs NIR photons and transforms them into thermal energy. Additionally, it generates NIR fluorescence within the 805–825 nm spectrum, rendering it appropriate for both in vitro and in vivo imaging studies. They also incorporated imiquimod (IMQ), a medication that stimulates the immune system and is used in the treatment of various cancer types, which activates antigen‐presenting cells. IMQ possesses significant promise for utilization as an immunotherapeutic agent through the stimulation of CD8^+^ T lymphocytes (CTLs) to combat cancer cells. It was anticipated that the resultant EpCAM–CS‐co‐PNVCL@IR780/IMQ NPs would facilitate the targeted release of medication at the tumor site. The integrity of the NPs will be preserved, as the neutral pH present in the systemic circulation will inhibit the dissolution of CS. When NPs are taken up by cells, the amide bond linking EpCAM to CS will be broken down by matrix metalloproteinases located in the extracellular matrix. Within tumor cells, the acidic conditions will lead to the dissolution of CS. Thus, IR780 and IMQ will be transported into the breast cancer cells. The composition is expected to initiate immunogenic cell death (ICD) of tumor cells upon exposure to NIR light, stimulate cytotoxic T lymphocytes (CTLs) that secrete cytokines such as Interferon Gamma (IFN‐γ), and restore the attachment of phospholipids to Acyl‐CoA Synthetase Long Chain 4 (ACSL4). By interacting with arachidonic acid, these phospholipids may instigate a cascade immunogenic ferroptosis.

#### 3.2.4 Apt‐Mediated Gene Therapy

Gene therapy is a medical approach that involving introduction of a genetic material, such as DNA or specific genes, into targeted cells through a vector in order to rectify genetic mutations within those cells. This method can serve to compensate for defective genes or facilitate the introduction of a normal gene copy to reinstate the proper function of the proteins [100]. This approach can be effectively applied to address numerous genetic disorders, such as cancer. Viruses can be altered to diminish their pathogenicity and subsequently used as carriers for the delivery of genetic material. Apts have proven to be applied in nonviral vector for the same purpose [101]. These molecules exhibit chemical stability and are resistant to denaturation, which enables a consistent chemical structure and potential for modification even postendocytosis. In addition, their nonimmunogenic properties and enhanced ability to penetrate tissues render them particularly suitable for therapeutic applications [102].

Osteosarcoma (OS) represents a highly aggressive tumor in children, characterized by extensive pulmonary metastases and pathological bone destruction [103]. Vascular endothelial growth factor A (VEGFA), which is significantly overexpressed in OS, promotes angiogenesis within the tumor microenvironment by paracrine stimulation of vascular endothelial cells [104, 105]. Additionally, it functions as an autocrine survival factor for the tumor cells themselves, making it a compelling target for therapeutic intervention in OS [106]. Clustered Regularly Interspaced Short Palindromic Repeats/Cas9 (CRISPR/Cas9) represents a versatile tool for genome modification that holds considerable promise for cancer treatment. Nonetheless, a primary obstacle in realizing the therapeutic capabilities of CRISPR/Cas9 is the absence of effective in vivo delivery systems that specifically target tumors. In 2017, Chao et al. [107] developed a lipopolymer known as PEG‐PEI‐CHOL (PPC), which was functionalized with LC09 and composed of PEG–PEI–cholesterol. This polymer was designed to encapsulate CRISPR/Cas9 plasmids expressing VEGFA gRNA and Cas9, following an assessment of an OS cell‐targeting Apt (LC09). Based on their research, LC09 facilitated the targeted delivery of CRISPR/Cas9 in both orthotopic OS and lung metastases. This resulted in effective genome editing of VEGFA within tumor cells, a reduction in VEGFA expression, suppression of lung metastasis and orthotopic OS severity, as well as a decline in angiogenesis and bone lesions, all without any noticeable toxicity. This approach to delivery may facilitate the application of CRISPR–Cas9 in the clinical management of cancer by concurrently blocking both autocrine and paracrine signaling of VEGFA in tumor cells.

In 2022, Khademi et al. [108] reported a direct approach to efficiently transport CRISPR/Cas9 into the nuclei of intended tumor cells, all the while mitigating off‐target effects. A versatile delivery system for the knockout of Forkhead box protein M1 (FOXM1) was developed by incorporating a cell‐targeting polymer (hyaluronic acid (HA)) along with a cell and nuclear targeting component (AS1411 Apt) onto the exterior of NPs that consist of a genome editing plasmid and CS serving as the core (Apt–HA–CS–CRISPR/Cas9). The results have been validated through cytotoxicity assessments and western blotting techniques. Analyses using flow cytometry and fluorescent imaging demonstrated that Apt–HA–CS–CRISPR/Cas9 was significantly taken up by the target cell lines (MCF‐7, SK‐MES‐1, and HeLa), while nontarget cells (HEK293) showed minimal internalization. In addition, in vivo studies demonstrated that Apt–HA–CS–CRISPR/Cas9 exhibited a significant tumor‐suppressive effect and effectively delivered CRISPR/Cas9 to the tumor site, showing no noticeable distribution in other organs in contrast to the unmodified plasmid. This approach illustrates the potential for creating targeted in vivo gene editing therapies with reduced side effects.

The EpCAM gene encodes a type‐I transmembrane glycoprotein that is found in elevated levels within various malignant epithelial cells and facilitates tumor proliferation by affecting the expression of several oncogenes, such as Cellular Myelocytomatosis (c‐myc) and additional cyclins [109]. In light of this association with tumorigenesis, the EpCAM gene has recently been recognized as a potential target for cancer therapy. In 2024, Sourav and colleagues [109] sought to eradicate the expression of proto‐oncogenic EpCAM by successfully administering a CRISPR plasmid through a lipid NP system made up of synthetic stimuli‐sensitive lipids. The plasmid contains crucial information presented as a guide RNA specifically designed for the EpCAM gene. The Apt‐functionalized approach precisely targets cells that overexpress EpCAM and successfully inhibits genetic expression. This study explored the pH‐responsive characteristics of the produced lipid NPs and evaluated their effectiveness in cancer cell lines derived from various sources exhibiting elevated levels of EpCAM. The occurrence has also been validated in vivo using tumor models in nonimmunocompromised mice. In summary, the recently developed method involving Apt‐coated lipid NPs has shown to be efficient for the delivery of EpCAM‐targeted CRISPR/Cas9 plasmid.

### 3.3 Limitations of Apt Application

Apts, albeit being small and easily penetrating biological barriers, may contribute to heightened renal filtration. This phenomenon could diminish the effectiveness of the therapeutic intervention. Other limitations include serum stability, and the ability to evade endocytosis. Nevertheless, it is feasible to address these challenges [110]. For example, Apts may be employed alongside higher molecular weight compounds to counteract excessive filtration in the kidneys. The issue of stability in serum can be tackled by modifying the backbone of the nucleic acid and implementing changes at the 5ʹ or 3ʹ terminus [111]. The creation of polymerases featuring mutations may arise from alterations in the bases that enhance the accessibility of different Apts. Such polymerases have the potential to enhance Apts that are unable to evolve naturally [112]. Through the development of peptides that facilitate endosomal escape based on Apts, it becomes possible to regulate the process of endosomal escape. NPs that are linked to Apts can effectively address the drawbacks associated with the use of Apts in isolation.

## 4 Applications of Antidotes

### 4.1 Anticoagulation Treatment

The complex procedure of coagulation involves the interaction of tissue‐based proteins with circulating cells and coagulation factors to create an insoluble clot at the site of arterial damage [113]. The process of blood clotting, while beneficial in response to specific vascular issues such as heart attacks and strokes, can sometimes be unwanted [114]. This occurs when clot formation takes place within the coronary or cerebrovascular beds [115]. In cardiovascular diseases, certain coagulation factors such as factor IX are crucial in the clotting process. In addition, inhibitors are utilized to prevent blood clot formation [116]. However, it is essential to monitor the effectiveness of anticoagulant medications closely to avoid potential severe side effects [117]. For example, one of the most commonly performed surgeries globally is coronary artery bypass graft surgery, with the frequent utilization of cardiopulmonary bypass (CPB) during the procedure [118, 119]. A patient undergoing CPB requires a high level of anticoagulation in the bloodstream. Historically, heparin has been the primary anticoagulant used for CPB procedures, with protamine available to reverse its effects postsurgery [120]. Regrettably, there are undesirable side effects linked to the therapeutic uses of protamine and heparin [121, 122]. Patients with CPB who are treated with heparin release a significant number of active enzymes, specifically thrombin, leading to a notable inflammatory response in the body [123, 124, 125]. The administration of heparin can potentially lead to heparin‐induced thrombocytopenia (HIT), affecting around 5% of patients, and triggering the production of antibodies [126]. A recent discovery has demonstrated the ability of an anticoagulant Apt‐complementary sequence combination to specifically inhibit factor IX activity, offering a potential alternative to traditional drugs with reduced side effects [127].

The initial drug–antidote combination developed using this approach and tested in humans is the REG1 anticoagulant system, studied by Dyke et al. in 2006 [128]. It consists of RB006, a unique Apt‐derived blocker of clotting factor IXa, and RB007, an antidote developed to counteract the pharmacological effects of RB006. RB006 binds strongly and specifically to coagulation factor IXa as a direct inhibitor. RB007, an oligonucleotide, effectively binds to RB006 and neutralizes its antifactor IXa activity. Preclinical studies have demonstrated that RB007 has the ability to swiftly and enduringly counteract the anticoagulant effects of RB006 when administered intravenously [129, 130].

**FIGURE 4:**
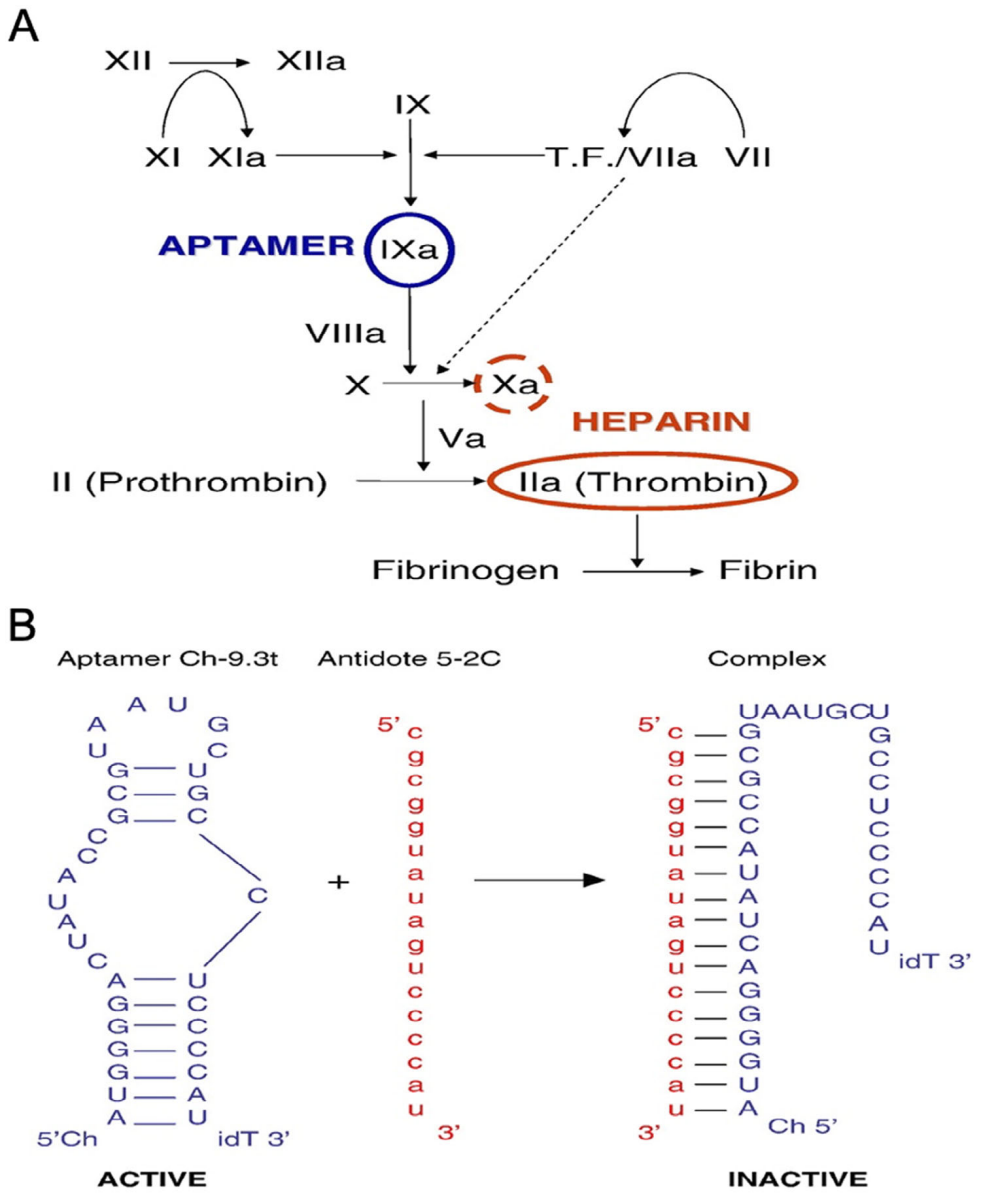
The FIXa aptamer 9.3tC is capable of facilitating CPB. (A) This coagulation cascade illustrates how aptamer 9.3tC selectively inhibits FIXa (blue) whereas heparin (red) inhibits thrombin and FXa. (B) Anti‐FIXa aptamer's predicted secondary structure, and its interaction with antidote 5‐2C to regulate aptamer function [129]. Copyright 2006, Cell Press.

In a separate research study, Nimjee et al. [129] explored the idea that reducing thrombin production and inflammation during CPB could potentially enhance heart function. They proposed the utilization of an anticoagulant‐antidote combination that targets the coagulation cascade at a point before heparin–protamine action (which does not affect thrombin) [129]. The initial generation of thrombin and the subsequent amplification of the coagulation cascade are primarily reliant on FIXa (Figure 4) [131, 132]. According to this study, anticoagulants that focus on the factor VIIIa–factor IXa complex could be helpful for treating CPB. To test this, a study was conducted using a porcine model of CPB to evaluate the effectiveness of an RNA Apt–antidote combination targeting factor IXa as a replacement for heparin–protamine. They found that the FIXa Apt‐complementary sequence combination is superior to heparin in controlling thrombin production and inflammation during CPB, as well as maintaining the patency of the CPB circuit and minimizing bleeding after surgery.

Therefore, the oligonucleotide antidotes can effectively counteract the impacts of the factor IXa Apt, while avoiding any negative side effects. Additionally, they have the potential to drive advancements in biology‐driven, adjustable, and quickly reversible treatments with enhanced safety and pharmacological characteristics.

In 2023, Nagano et al. [133] discovered an antithrombin DNA Apt named M08s‐1, which shows promise as an anticoagulant. This bivalent Apt has a better safety profile when used with an antidote. In comparison with individual monomers, this Apt demonstrated a binding affinity to human and mouse thrombin that was roughly 100 times greater. Protamine sulfate was employed as a countermeasure against the highly potent bivalent Apt in this study. The findings revealed that an antidote could effectively nullify the function of the Apt. Additionally, protamine sulfate and the antidote successfully inhibited the activity of the M08s‐1 Apt. This procedure is advantageous for enhancing the safety of drug development in the treatment of HIT. Therefore, the discovery of potent and neutralizable anticoagulant Apts holds promise for HIT therapy, offering an improved safety profile [133].

Hence, the effects of the factor IXa Apt could be promptly nullified by the oligonucleotide antidotes without causing adverse reactions. Additionally, they could function as a potent driver for developing rapidly reversible, adjustable, bioengineered treatments with improved safety and pharmacological characteristics.

**FIGURE 5:**
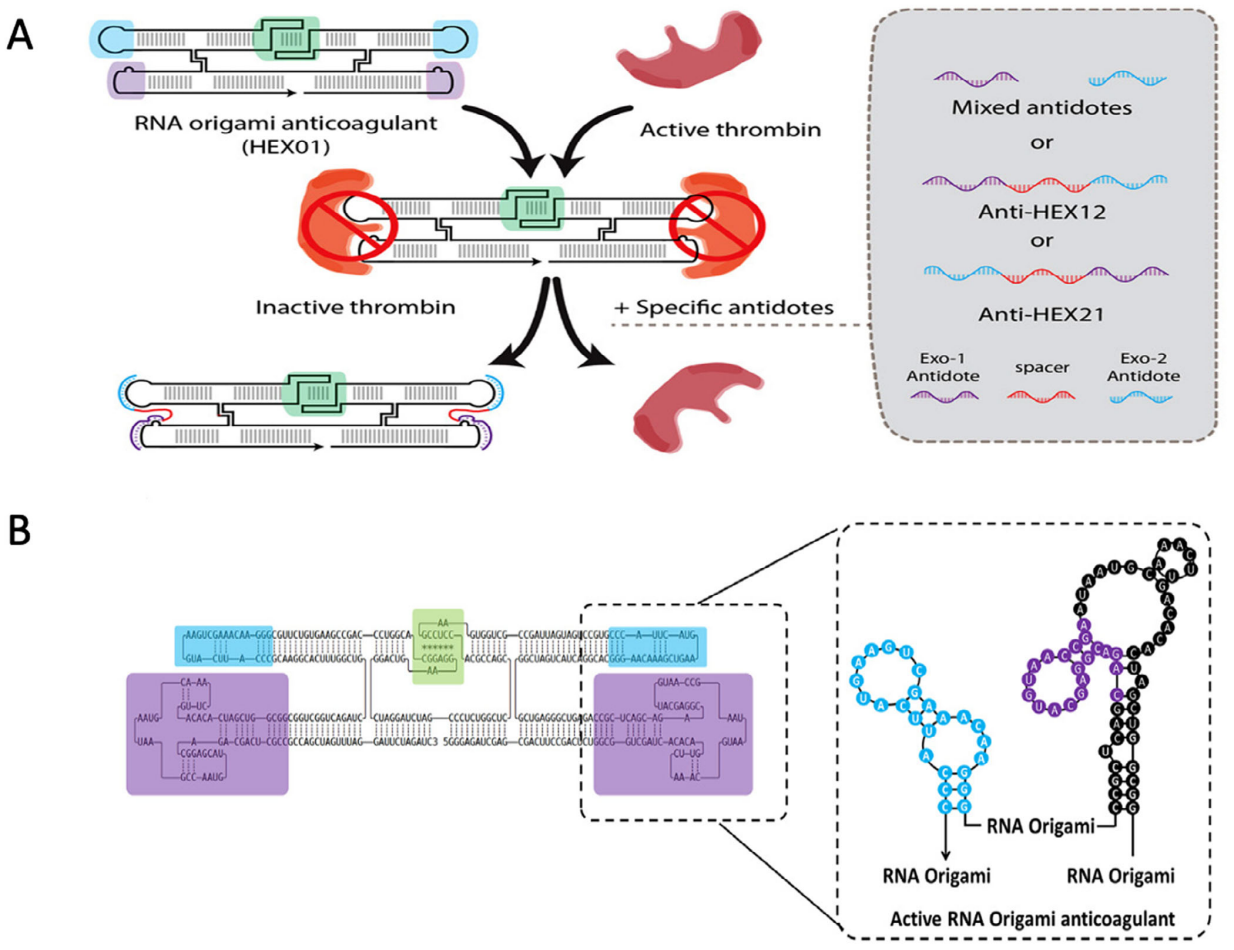
The mechanism of single‐molecule DNA antidotes reversal activity and scheme of thrombin‐binding RNA aptamer attached to RNA origami anticoagulant. (A) Schematic illustration of RNA origami anticoagulant (HEX01) interacting with single‐molecule DNA antidotes. The inset illustration shows the composition of mixed antidotes and single molecule DNA antidotes with and without spacers.(B) Two‐dimensional illustration of multivalent, thrombin‐binding RNA aptamers appended on RNA origami anticoagulant [134]. Copyright 2024, Molecular Therapy.

Intravenous anticoagulants are commonly applied in medical treatments to minimize unexpected blood clotting. However, some of these anticoagulant therapeutics lack effective reversal agents and exhibit concerns regarding safety. In 2024, Krissanaprasitet al. [134] introduced new findings regarding a RNA origami‐based direct thrombin inhibitor (HEX01). HEX01 possessed multiple thrombin‐binding Apts attached to the RNA origami (Figure 5). It displayed significant anticoagulant function in both in vitro and in vivo. Whole‐mice HEX01 biodistribution studies utilizing ex vivo imaging revealed that it accumulates mainly in the liver and 10‐fold less in the renal system. Following this, they reported a new, swift reversal agent (antidote) and presented the initial in vivo biodistribution findings regarding the reversibility, efficacy, and preliminary safety of this compound. This single‐molecule DNA antidote (HEX02) reversed HEX01's anticoagulant performance in human plasma within a minute and was well functioned in a mouse model. In addition, these results demonstrated that the HEX01/HEX02 system is neither cytotoxic to epithelial cell lines nor hemolytic in vitro. Furthermore, they observed no serum cytokine reaction to HEX01/HEX02. Thus, HEX01 and HEX02 were deemed a safe and efficient system for coagulation management, exhibiting significant promise for prospective medical uses.

### 4.2 Cancer Treatment

There is a pressing need for treatments that can eliminate both primary and secondary tumor growths without causing harm to healthy tissues [135]. Current remarkable oncology advancements in cancer treatments originated from an ancient concept: ADCs [136]. Most of the substances targeted by antibodies and ADCs are not exclusive to cancer cells, but are also found in low levels in healthy tissue [137]. Consequently, antibody‐based therapies have been associated with side effects caused by the build‐up of antibodies in noncancerous tissues [138]. While antibodies are challenging to modify and require humanization, Apts have been discovered to target molecules with distinctive and beneficial properties compared with antibodies, including simpler chemical synthesis and customization [139].

Cis‐diamminedichloroplatinum (II) (cisplatin) is an ideal chemotherapeutic agent for the broad range of cancerous tumor therapy [140]. Even though cisplatin has remarkable effectiveness against cancer, its major drawbacks include lack of selectivity and negative side effects. Lu et al. [141] discovered the creation of controlled formulations of Apt (AS1411 Apt)–liposome conjugate and cisplatin‐encapsulated multifunctional liposomes. The utilization of this delivery system demonstrates specific targeting of cancer cells and drug delivery. They also suggested for the first time that the Apt's complementary DNA (cDNA) could potentially be used as a countermeasure to interfere with Apt‐mediated targeted drug delivery. This reversible delivery approach is suitable for a wide range of chemotherapeutic pharmaceuticals. The creation of the versatile Apt–liposome–cisplatin complexes was straightforward and reproducible. Also, the utilization of the Apt‐coated liposome drug‐delivery platform proved to be far simpler compared with an antibody‐based method. The antidote cDNA can quickly stop and dismantle the binding of the Apt to the receptor on the cell surface. This discovery provides further support for the high selectivity of an Apt‐based cancer‐targeting method, which could be tailored for specific drug delivery purposes.

**FIGURE 6:**
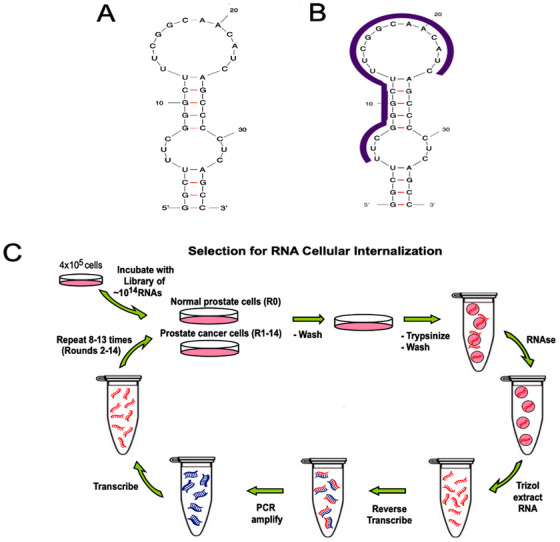
Graphical abstract of Cell‐Internalization SELEX against prostate cancer cells. (A) The mfold software was used to predict the secondary structure of the 36‐nt variant of the E3 aptamer. (B) The antidote oligonucleotide targets the region of the E3 aptamer shown in purple. (C) The E3 aptamer, which was identified through Cell‐Internalization SELEX against prostate cancer cells, has been reduced to 36 nt [142]. Copyright 2018, PNAS.

In a study conducted in 2017, Sullenger et al. [142] aimed to develop a new targeted therapy for prostate cancer by applying Cell‐Internalization SELEX to discover the E3 Apt (Figure 6C). This Apt was found to enter prostate cancer cells specifically, while leaving normal cells unaffected. Through the chemical attachment of E3 Apt to potent cytotoxins such as auristatin drugs MMAE and MMAF, they successfully created toxic compounds that effectively targeted and killed prostate cancer cells (LNCaP, PC‐3, DU 145, and 22Rv1). The MMAF–E3 conjugate has shown a precise method for treating individuals with prostate cancer. Furthermore, E3 RNA Antidote 5 (20‐nt) successfully inhibited the function of the E3 Apt (Figure 6B), thus hindering cell attachment and uptake. These antidotes were also able to counteract the harmful effects of the ADCs. Surprisingly, an excess of E3 antidote twice the normal amount effectively counteracted the effects of both drug conjugates (MMAF–E3 and MMAE–E3), thereby halting cell death. The findings of this study suggest that the E3 Apt can be regulated and deactivated through Apt‐complementary sequence intervention in case of any adverse reactions, despite its specific targeting of cancer cells.

Leukemia, a type of cancer affecting bone marrow and blood cells, is the one of leading causes of death, particularly in children [143]. Chemotherapy, the primary treatment for acute lymphoblastic leukemia (ALL), often involves the use of daunorubicin (Dau), an anthracycline antibiotic. However, the clinical use of Dau is limited due to its cumulative cardiotoxicity, especially in pediatric patients [144, 145]. In order to tackle this issue, the use of nanocarriers for delivering anticancer medications has become a promising method to enhance drug effectiveness and mitigate unintended side effects [146]. In 2011, Taghdisi et al. [147] demonstrated the use of a versatile Apt system to create a connection between Dau and single‐walled carbon nanotubes (SWNTs) to improve the targeted delivery of Dau to ALL T‐cells (Molt‐4). The release rate of Dau in this system is influenced by the pH level of the surrounding environment. At pH 5.5, the drug release rate was enhanced approximately six‐fold. Furthermore, the use of antisense oligos against the Apt can effectively hinder the transfer of Dau to Molt‐4 cells. Research on the antisense‐dependent cell survival revealed that the sgc8c antisense successfully inhibited the uptake of the Dau–Apt–SWNTs tertiary complex by Molt‐4 cells and reduced cell death. Their findings also indicated that treating cell cultures with the Dau–Apt–SWNTs complex and three times the dose of antidote could boost cell viability to 90%.

**FIGURE 7:**
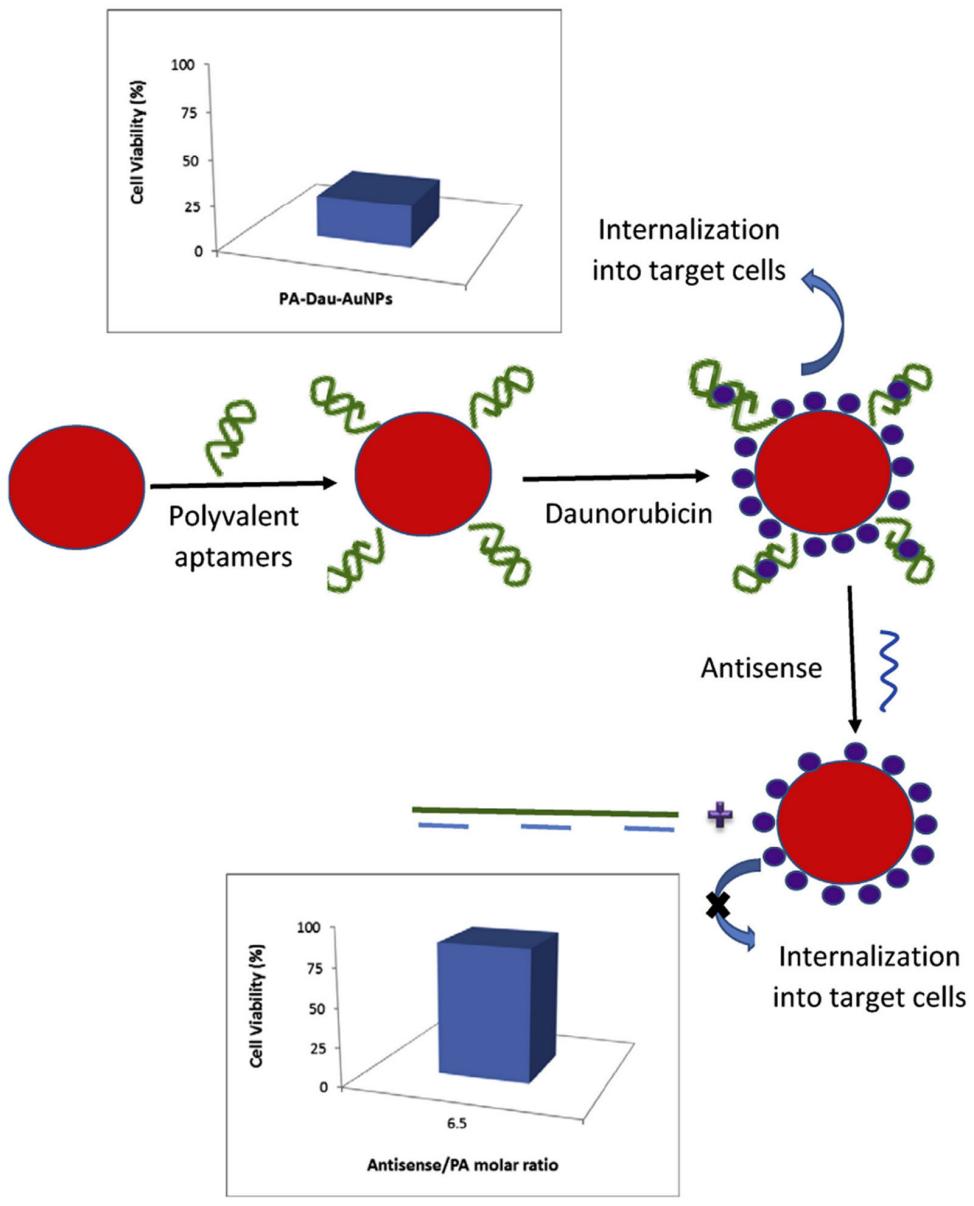
Graphical description of the synthesis of the PA–Dau–AuNPs complex and the antisense mechanism. Polyvalent aptamer blocks attached to AuNPs, enabling a rapid and effective reversal of the cytotoxic effects of the PA–Dau–AuNPs complex utilizing the antisense of the polyvalent aptamers [148]. Reproduced with permission from reference. Copyright 2016, Elsevier.

In another study, Taghdisi et al. [148] successfully developed a modified polyvalent Apt (PA)–Dau–AuNPs complex in a project (Figure 7), demonstrating its efficacy on Molt‐4 cells, a human ALL T‐cell line. The modified PA system used to enhance Dau's therapeutic potential included two types of Apts: sgc8c and AS1411. The PA–Dau–AuNPs complex effectively loaded Dau (10.5 µM) onto PA‐modified AuNPs. Notably, Dau was discharged from the compound based on pH levels, showing a quicker release at pH 5.5. Flow cytometry analysis confirmed the successful uptake of the PA‐Dau‐AuNPs compound by target cells (Molt‐4), while nontarget cells remained unaffected. This approach led to an improved therapeutic outcome of Dau. Additionally, PA blocks were linked to AuNPs via electrostatic interaction, facilitating the rapid and efficient reversal of cytotoxicity induced by the PA–Dau–AuNPs complex with the help of antisense PAs. Further, the toxic effects of the PA–Dau–AuNPs complex can be effectively neutralized by employing antisense of PA. The compatibility with living organisms and large surface area of AuNPs make them ideal carriers for transporting drugs. This strategy ensures precise delivery of the medication to the desired location, reducing unintended side effects. In contrast to their previous research utilizing a single Apt (sgc8c), the current study utilized a modified PA system incorporating two different Apts (AS1411 and sgc8c Apts), resulting in an enhanced therapeutic efficacy of Dau. Additionally, in this investigation, clusters of PAs were affixed to AuNPs via electrostatic interactions, enabling the rapid and efficient reversal of cytotoxicity in the PA–Dau–AuNPs complex applying antisense PAs. Overall, this project underscores the potential of targeted drug delivery through nanocarriers to enhance the effectiveness and safety of anticancer medications.

In 2020, Zhang et al. [149] designed a novel Apt‐based delivery system for neuroblastoma (NB) treatment. They proposed a pH‐responsive, adjustable drug delivery system referred to as “IGD‐Target,” which utilizes the i‐motif, Apt, and linker. This system has the capability to distinguish between the target expression found on normal cells and carcinoma cells. They demonstrated that the combination of an Apt (GD2 Apt) and i‐motif represents a dependable strategy for cancer therapy, particularly when the target is also found on healthy cells, suggesting considerable clinical promise for various cancer treatments. In this framework, the i‐motif is engineered to adjust its allosteric properties within the acidic microenvironment of tumors, enabling the Apt to specifically identify GD2 present in cancerous cells while remaining unresponsive to normal cells that exist in a neutral setting. As a result, it facilitates precise targeting of Dox to malignant cells and safeguards healthy tissues. The research additionally presented in vivo experiments that illustrated the capacity of IGD‐Targeted to suppress tumor proliferation and diminish adverse effects in comparison with conventional chemotherapy. The outcomes also examined the molecular and cellular processes that contribute to the therapy's efficacy. In summary, the results indicate that IGD‐Targeted may represent a promising approach for targeted cancer treatment, especially concerning NB.

### 4.3 Imaging

New and innovative compounds with great potential for molecular imaging continue to be researched and created, increasing the range of agents that can be used in clinical settings and promoting the development of new imaging techniques [150]. Apts, such as those detectable by MRI, PET, and FRET, represent a type of probe that integrates multiple imaging reporters simultaneously. This approach allows for the direct visualization of a blood clot itself, rather than relying on the absence of blood flow as an indicator [151]. Molecular imaging of thrombosis necessitates a probe capable of circulating in the bloodstream and penetrating the clot [152]. It is advantageous for the probe to have a short circulation half‐life to facilitate rapid clearance of unbound probes. DNA and RNA Apts exhibit high affinity and specificity for their targets [153]. Due to their small size and brief circulation times, they possess favorable pharmacokinetic properties for imaging purposes. As with antibodies, Apts display strong binding to their targets, but they can be chemically synthesized and modified to suit specific needs [154]. Notably, Apt activity can be promptly altered by applying short complementary oligonucleotide that bind to the Apt and influence its conformation [37].

The process of blood clotting, known as thrombosis, can block important blood flow to organs in the body, leading to serious cardiac issues such as ischemic stroke, pulmonary embolism, and myocardial infarction [155]. In order to address these issues, Layzer et al. [150] used a combination of Apt and antidote to enhance patient outcomes by quickly restoring blood flow. This combination acts as a dynamic binding agent, contrast agent, and rapid clearance‐inducing probe for the crucial clotting factor thrombin. In its role as a molecule designed to target thrombin, Tog25t successfully detected the formation of blood clots and efficiently identified preexisting clots in the jugular and femoral veins of mice through fluorescence imaging. Due to thrombin's specific distribution limited to sites of active clotting, Tog25t specifically binds to thrombin without interacting with prothrombin or thrombin‐antithrombin complexes. The research team conducted a thorough analysis and enhancement of Tog25t thrombi screening by linking the Apt with the NIR dye (AF680). They illustrated that a combination of Apt and Apt‐complementary sequence can be utilized to trigger and stop clot imaging in mice by circulating AF680–Tog25t conjugate to thrombin for precise identification of jugular blood clot formation in an antidote‐reversible process. Following this, the administration of an antidote accelerated the detection of the clot by eliminating AF680–Tog25t from the bloodstream, resulting in a clear and quick visualization of the clot‐bound AF680–Tog25t. As a result, a thrombus imaging agent that is reversible and adjustable was created, leading to faster imaging.

### 4.4 Cell Isolation

The exploration and analysis of individual cells are increasingly impacting various fields within the life sciences and biomedical research, particularly in relation to a wide range of cell groups, including those found in numerous types of cancer [156]. One method for physically separating individual cells from surrounding materials and/or from one another is through the use of single‐cell isolation techniques [157]. In biotechnology and microbiology, single‐cell isolation methods serve three primary purposes: (1) cultivating bacteria that have not been grown previously; (2) assessing and monitoring the function and behavior of cells; and (3) discovering novel microbiological products such as antibiotics and enzymes. Numerous methods for isolating single cells have been developed, including compartmentalization, micromanipulation, flow cytometry, dilution, and microfluidics [158, 159]. Reversible Apt–antidote couples provide a novel strategy in this field [160]. A genuine Apt designed for a specific target will bind based on its unique structure; a precise Apt will strongly attach to its intended target [161]. Utilizing short complementary oligonucleotides to complex with Apts allows for the development of reversible Apt‐complementary sequence that act in an antisense manner to a specific section of the Apt [162]. These countermeasures function by reversing the attachment of the Apt to its target through pairing with a matching section of the Apt, thus altering its shape from an active to an inactive form [163, 164]. Cancer cell populations, such as circulating tumor cells (CTCs), exhibit diversity [165]. CTCs are uncommon cancer cells released into the bloodstream from primary and metastatic tumor locations, whether they are living or dying [166]. As a result of this intricate nature, only a restricted range of techniques have been developed for the isolation of particular CTC subgroups [167]. Due to this intricacy, only a limited number of methods have been devised to separate specific CTC subpopulations. These techniques include gradient centrifugation, di‐electrophoresis, mRNA tagging, size‐based exclusion, and affinity‐based enrichment [168]. In 2016, Green et al. [168] Cintroduced a novel technique involving Apts and a fluidic chip to separate different subpopulations of cancer cells in two dimensions (2D). The approach involves using DNA Apts that are designed to target cell‐surface markers in order to capture the cells, and then releasing them by using complementary antisense oligonucleotides. The research concentrated on specimens from individuals receiving therapy for advanced prostate cancer, categorizing them according to EGFR and EpCAM‐directed Apts [169]. Ultimately, the researchers demonstrated the effectiveness of the 2D sorting technique in analyzing samples from cancer patients [170].

**FIGURE 8:**
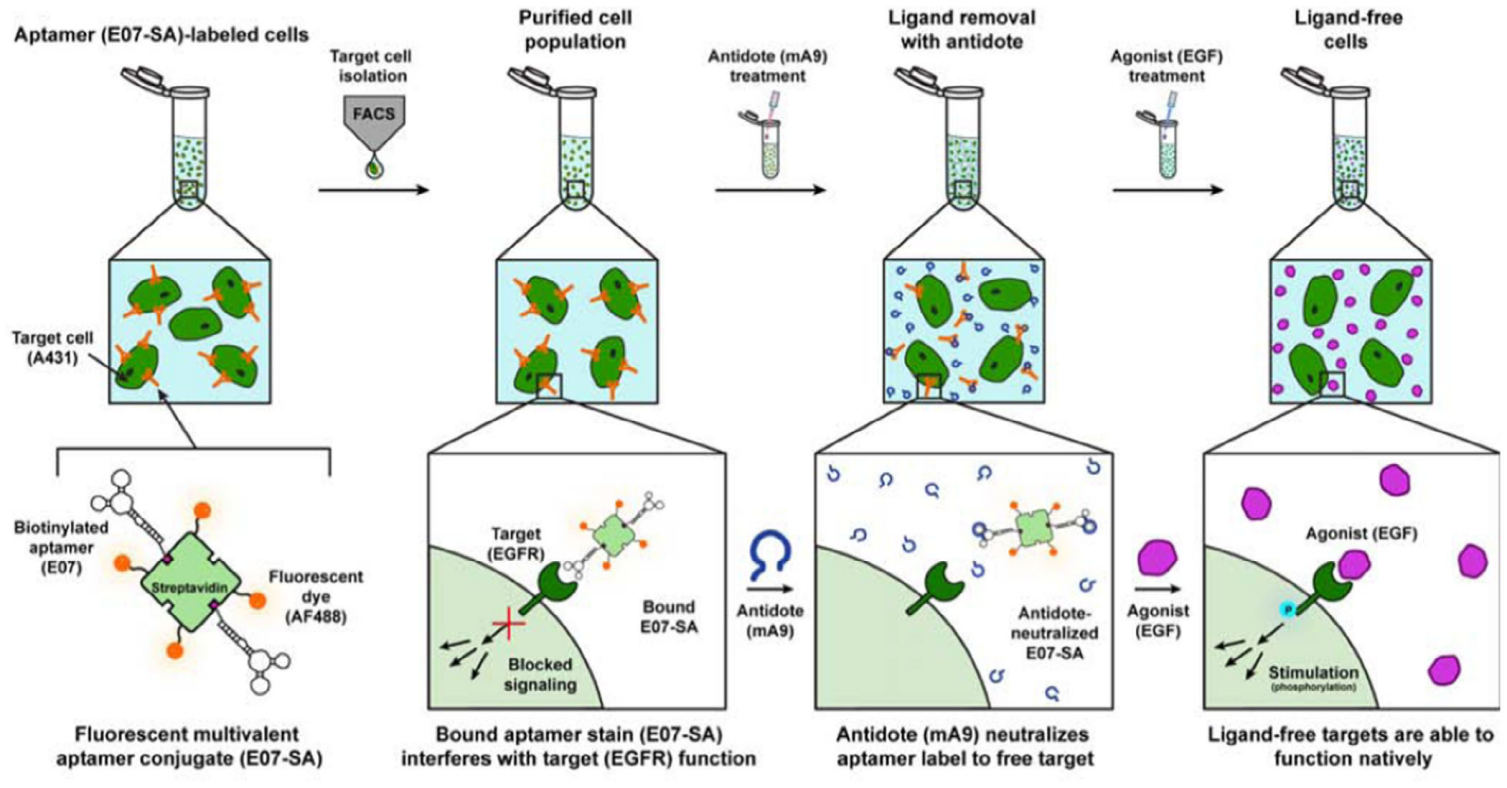
Through the use of an antidote, aptamers can reverse their attachment to cell surfaces, allowing for the efficient purification of cells and the production of cells in their natural state. The EGFR(+) cells were initially stained with the green fluorescent AF488‐SA‐E07 and subsequently sorted based on the E07/AF488+ signal. Then, an antidote treatment using mA9 was applied to remove the E07 stain, resulting in a population of ligand‐free unlabeled cells [135]. Copyright 2020, Cell Press.

Gray et al. [135] created a two‐part system that included a fluorescent dye‐Apt and Apt–magnetic bead combinations to specifically mark cells for purification (Figure 8). They employed a corresponding antidote to remove the E07‐Apt and restore the EGFR (+) cells to their original condition. This advancement will greatly improve the effectiveness of an Apt–antitoxin system for labeling and purifying cells prior to returning them to their natural state. The key discovery was that antidote therapy successfully eliminated the E07 Apt, which attaches to EGFR (+) cells, from isolated cells. This allows the cells to return to their original state and resume normal EGFR function. The combination of E07 and antidote acts as a molecular switch that controls cell labeling by toggling Apt binding, antidote treatment, and Apt blocking. Last, it was noted that a 99% pure group of desired cells can be isolated from diverse mixtures of cells with high complexity [135].

The potential to undo the attachment of Apts to their specific proteins has garnered significant interest in the development of adjustable therapeutic treatments [85]. Lately, there has been a growing interest in utilizing Apts as reversible ligands for cell sorting [171]. Currently, antibodies are employed to isolate cells that express a specific cell surface receptor. Nevertheless, the incapacity to separate antibodies from sorted cells severely hinders their effectiveness for a variety of purposes [172]. In a study conducted in 2024, Requena et al. [161] highlighted the current practice of using specific Apt‐complementary sequences to remove fluorescent Apts during fluorescent‐activated cell sorting (FACS) studies. They demonstrated that this method is relevant to a wider spectrum; Apts are capable of identifying various cell types at the same time during FACS analyses. Additionally, they introduced a new method to reverse Apt binding after cell sorting by utilizing a nuclease. This technique provided an efficient approach for FACS, removing the necessity of generating antidote oligonucleotides for individual Apts. This method will considerably reduce expenses and improve the effectiveness of Clean FACS.

A new method called toehold antidote has been introduced as a potential solution for cell isolation and addressing the challenges mentioned [173, 174]. In this approach, a toehold region is added to one end of the Apt to improve the binding with the Apt‐complementary sequence [6, 175, 176]. Essentially, the antidote attaches to both the toehold region and the Apt segment in this scenario. Toeholds are short sequences of single strands that enable the binding of complementary sequences and the replacement of prepaired bases by a process called strand displacement [177]. In this scenario, the reversing agent would engage in strand displacement through the toehold to disrupt the intra‐strand base pairing in the Apt necessary for its secondary configuration [178]. In 2019, Kacherovsky et al. [179] demonstrated that DNA Apts, produced using a modified cell−SELEX method to exhibit high affinity in the low‐nanomolar range for the T‐cell marker CD8, allow for the efficient and cost‐effective isolation of pure CD8+ T cells without leaving any trace. The isolated CD8+ T cells were liberated without the need for labeling by using complementary oligonucleotides that engage in toehold‐mediated strand displacement with the Apt. A reversal agent was specifically developed by the researchers to facilitate the label‐free release of the captured CD8^+^ T cells, achieved through a 36‐base complementary oligonucleotide reversal agent. This antidote was created with the intention of interfering with the structure of the CD8‐specific Apt, enabling the CD8+ T cells to be freed from the Apt‐immobilized supports with a high level of purity and yield. It has also been shown that chimeric antigen receptor T cells derived from these cells display comparable traits to antibody‐purified chimeric antigen receptor T cells in terms of proliferation, morphology, functionality, and efficacy in fighting tumors in a mouse model of B‐cell lymphoma. Through the utilization of several Apts and their complementary oligonucleotides, Apt‐mediated cell selection has the potential to facilitate the complete artificial, step‐by‐step, and undetectable isolation of specific lymphocyte subgroups from a unified system.

In general, the benefit of being able to reverse the effects with an antidote may still be present when utilizing Apts for cell labeling and purification instead of antibodies. Therefore, in techniques for isolating cells, the actions of Apts on cells can be undone, allowing them to revert back to their initial state by generating unlabeled cells once the Apt tags are eliminated.

### 4.5 Viral Infection

Nucleic acid‐derived Apts represent a versatile instrument for biological regulators [180]. To efficiently utilize Apt regulators in nonequilibrium molecular networks, the interaction between Apt and ligand needs to be switchable over time [181]. For this, the antidotes as adjustable elements can assist in the suppression or activation of certain processes such as viral infections [182].

In 2018, Lloyd et al. [183] indicated a simple technique could effectively inhibit viral RNA polymerase and dynamically control ligand–Apt binding. This group demonstrated the concept of employing “light‐up” RNA Apts, which are chosen to attach to particular small molecules and trigger their fluorescence. They began their project by investigating over function of Broccoli Apt, which binds to DFHBI, a structural replica of the Green Fluorescent Protein fluorophore (HBI). DFHBI turns luminous only when attached to its Apt. First, they designed DNA Apt‐displacing sequences (kleptamers), which are complementary to the Broccoli Apt, reducing the fluorescence of the sample and modulating the ligand–Apt conjugate fraction. They also explored whether strand Apt's displacement from their target could be used to regulate a biological process, such as in vitro transcription utilizing viral RNA polymerases (RNAPs), often employed in synthetic biology. For this, they utilized RNA Apts engineered to prevent the transcription activity of T7 and SP6 RNA polymerases. Kleptamers, which replace the RNAP‐inhibiting Apt in the inactive Apt–RNAP complex, are expected to activate the RNAP transcription function. To track and evaluate the efficiency of kleptamer activity, they utilized the Broccoli fluorescent Apt (as a reporter), with transcription controlled by a synthetic template containing either the T7 or SP6 viral (bacteriophage) promoters. They evaluated the fraction of active RNA polymerase as a function of Apt kleptamer concentrations using normalized kinetic data for Broccoli transcription. Computational simulations suggested that the RNAP Apt–kleptamer under consideration here is ideal for the development of nonequilibrium networks, which may be extremely resilient due to the ultrasensitive nature of stoichiometric Apt control. The efficacy of Apt displacement in medical treatments in vivo indicates Apt–kleptamer pairings could potentially be utilized straightaway to dynamically regulate cellular processes.

## 5 Conclusions and Outlook

Apts and their applications have significantly contributed to clinical diagnostics, cancer treatment, and gene delivery. The development and engineering of NPs utilizing Apts require enhanced creativity and efficiency. The remarkable synergy between the esteemed fields of nanotechnology and Apts has resulted in extraordinary advancements. A critical area that warrants further exploration in the future is the efficacy and in vivo application of these technologies. We are of the opinion that extensive discussions and thorough literature reviews are essential to further advance this groundbreaking technology. Ultimately, we believe that nanomaterials based on Apts will possess an even more promising future regarding their development and application.

The precise dosing and controllability of drug effects are crucial factors in reducing the negative effects of medication. Antidotes play a key role in reversing the effects of prescribed drugs by competitively altering, blocking, binding, or universally inactivating agents, showing great potential in minimizing adverse reactions [184, 185]. Apts, short single‐stranded chains of nucleotides capable of forming three‐dimensional shapes, have attracted medical interest because of their distinct characteristics. These include precise and strong binding to specific targets [33]. Additionally, the ability to modify Apts at specific sites offers the opportunity to adjust their availability and stability, making them applicable across various industries [186]. By modifying the three‐dimensional configuration of the Apt, such as by introducing a complementary oligonucleotide chain that pairs with the Apt, it becomes feasible to effectively hinder the binding of the Apt to its target. Hence, nullifying the ability of an Apt to attach to its target through the incorporation of a complementary oligonucleotide sequence can be regarded as a significant characteristic of Apts [163, 187, 188]. While using Watson‐Crick base pairing rules to create Apt‐complementary sequences can successfully alter Apt function, this approach is almost costly and time consuming due to the need for custom antidotes for each new Apt [189]. Furthermore, the formation of double‐stranded Apt–antidote oligonucleotide (dsRNA) complexes can trigger an innate immune response by activating toll‐like receptors. To address this issue, both the antidote oligonucleotide and the RNA Apt are frequently made up of modified oligonucleotides (such as 2´‐OMe RNA and 2´‐F). Nevertheless, given the documented instances of immune system activation by short 2´‐OMe siRNA duplexes, the concern regarding immune stimulation still remains [190, 191]. Hence, an essential strategy is required to address these constraints in antidote development. Creating universal antidotes emerges as a highly viable solution, capable of modulating the activity of numerous Apts irrespective of their specific sequences [192, 193]. In contrast to the specific pairing required for oligonucleotide antidotes, a universal antidote works by enveloping the Apt to block its binding to the target, creating a protective barrier. Essentially, the universal antidote functions like a “molecular sponge,” capturing and isolating each oligonucleotide administered to exert its effect through binding. Hence, potential candidates for a universal counteractive agent seem to possess several key characteristics: (1) the counteractive agent needs to have the ability to attach to every Apt with high affinity, irrespective of its folded structure or primary sequence. (2) Counteractive compounds should be nonimmunogenic and nontoxic similar to Apts. Last, the resulting counteractive‐Apt complex has to exhibit the required stability to inhibit the re‐release of the Apt. A potential universal antidote candidate is a positively charged porphyrin capable of attaching to G‐quartets, which are specific DNA structures composed of four guanosines arranged in a planar array. This antidote candidate is effective for Apts containing G‐quartets, such as the anticoagulant Apt ARC‐183, making it suitable for a subset of Apts with this structural feature [194]. An optimal solution for creating universal antidotes could involve a composite that can recognize a shared characteristic among all Apts. Given that Apts are oligonucleotides, which are inherently negatively charged, a positively charged compound that can interact with the Apt through electrostatic forces would be highly effective. Protamine, due to its positive charge and ability to bind to nucleic acids, has been shown to effectively neutralize the function of Apts [195]. Nevertheless, protamine is associated with well‐known adverse reactions including pulmonary hypertension, systemic hypotension, anaphylaxis, and as such, it should be utilized with extreme care [196, 197]. In a broad sense, the universal antidote approach is likely to be more universally applicable than the personalized antidote oligonucleotide technique. Antidotes, as with other medical treatments, need to be assessed based on factors including safety and stability. It is believed that the use of antidotes can be swiftly implemented in clinical settings, offering a new avenue for future therapeutic advancements.

## Author Contributions

Sepideh Hassibian, Mahsa Amin, Elham Sameiyan, and Reza Ghaffari wrote the manuscript and received permissions of figures. Seyed Mohammad Taghdisi, Khalil Abnous, and Seyed Mohsen Dehnavi contributed to the design of the manuscript structure and edited the final manuscript. Mohammad Ramezani and Mona Alibolandi assessed and reviewed the manuscript structure and ideas. All authors have read and approved the final manuscript.

## Conflicts of Interest

The authors declare that there are no potential conflicts of interest.

## Ethics Statement

The authors have nothing to report.

## Data Availability

The data that support the findings of this study are available in the manuscript.
